# Investigation of Singlet Fission–Halide Perovskite Interfaces

**DOI:** 10.1021/acs.chemmater.1c04310

**Published:** 2022-05-16

**Authors:** Alan R. Bowman, Samuel D. Stranks, Bartomeu Monserrat

**Affiliations:** † Cavendish Laboratory, Department of Physics, University of Cambridge, J.J. Thomson Avenue, Cambridge CB3 0HE, U.K.; ‡ Department of Chemical Engineering and Biotechnology, University of Cambridge, Philippa Fawcett Drive, Cambridge CB3 0AS, U.K.; § Department of Materials Science & Metallurgy, University of Cambridge, 27 Charles Babbage Road, Cambridge CB3 0FS, U.K.

## Abstract

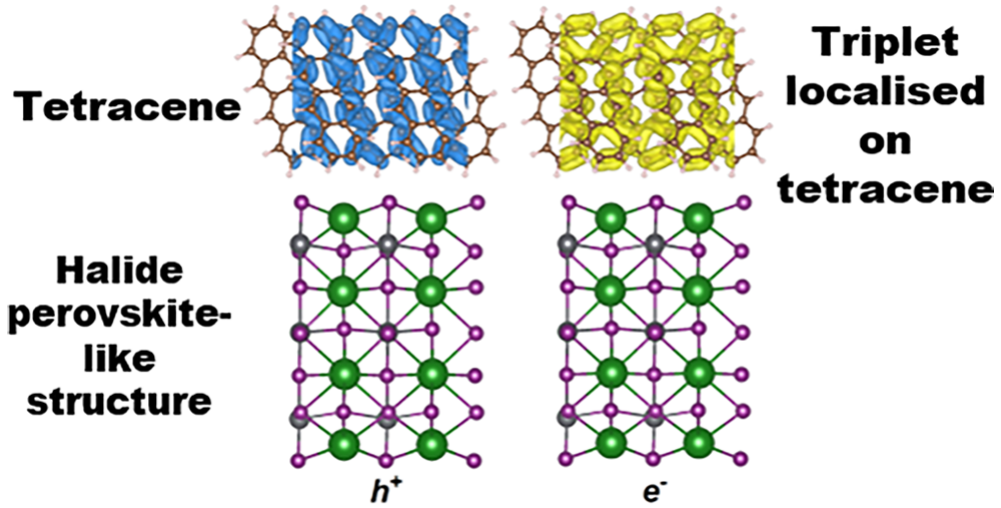

A method for improving the efficiency of solar cells is combining a low-band-gap semiconductor with a singlet fission material (which converts one high-energy singlet into two low-energy triplets following photoexcitation). Here, we present a study of the interface between singlet fission molecules and low-band-gap halide pervoskites. We briefly present 150 experiments screening for triplet transfer into a halide perovskite. However, in all cases, triplet transfer was not observed. This motivated us to understand the halide perovskite–singlet fission interface better by carrying out first-principles calculations using tetracene and cesium lead iodide. We found that tetracene molecules/thin films preferentially orient themselves parallel to/perpendicular to the halide perovskite’s surface. This result is in agreement with simulations of tetracene (and other rodlike molecules) on a wide range of inorganic semiconductors. We present formation energies of all interfaces, which are significantly less favorable than for bulk tetracene, indicative of weak interaction at the interface. It was not possible to calculate excitonic states at the full interface due to computational limitations, so we instead present highly speculative toy interfaces between tetracene and a halide-perovskite-like structure. In these models, we focus on replicating tetracene’s electronic states correctly. We find that tetracene’s singlet and triplet energies are comparable to that of bulk tetracene, and the triplet is strongly localized on a single tetracene molecule, even at an interface. Our work provides new understanding of the interface between tetracene and halide perovskites, explores the potential for modeling excitons at interfaces, and begins to explain the difficulties in extracting triplets directly into inorganic semiconductors.

## 1 Introduction

Material surfaces and interfaces are important in a range of technologies and govern processes including sample growth, ion mixing, charge transfer, and electronic passivation.^1−7^ An interface of particular interest for the next generation of solar cells is between a singlet fission material and an inorganic semiconductor. Singlet fission materials have the unusual property that when a singlet is generated (following photoexcitation) it is rapidly converted into two triplets.^8^ If combined with an inorganic semiconductor harvesting low-energy wavelengths of the solar spectrum, such a solar cell could surpass the Shockley–Queisser single junction efficiency limit of 33%.^9^ Key to this technology working is the ability to extract at least part of the triplet excitons from the singlet fission material. Ideally, triplets would be transferred directly into, or separated at, an interface with an inorganic semiconductor (exciton transfer and exciton dissociation). Triplet excitons cannot undergo Förster energy transfer,^10^ as they do not have a transition dipole moment (unlike singlet excitons). Therefore, charge transfer must proceed via Dexter processes, which are far shorter-ranged in nature and governed by wave function overlap.^11^

In studies of clean silicon surfaces with singlet fission materials deposited on them, the component of triplet transfer has been negligible.^12,13^ However, extraction of triplets has been achieved into PbS and PbSe quantum dots, as well as more recently silicon.^14−17^ For extraction into PbS and PbSe, direct chemical bonds were formed between the organic molecule and the inorganic semiconductor, while for the case of silicon, an interlayer between the two materials was required to facilitate triplet transfer. More generally, it has proven difficult to extract triplets directly from singlet fission materials into inorganic semiconductors, as is highlighted in several studies.^12,13^ A range of processes may prevent triplet charge transfer, including transport of excitons to the interface being hindered, defect sites trapping triplets at the interface, poor quality semiconductor surfaces (i.e., due to contaminants), or the formation of interfacial states which cannot disassociate.

Here, we explore the interface between singlet fission materials and halide perovskites. First, we briefly present 150 experiments screening for triplet transfer from singlet fission materials to low-band-gap (<1.25 eV) halide perovskites. Despite modified fission activity in the organic, a range of different exposed surfaces, varying triplet energies, and changing the inorganic material, in all cases, we observed singlet transfer or no transfer to the halide perovskite. These experiments motivated us to understand the interface better via a first-principles computational study. We modeled the interface between the singlet fission material tetracene and cesium lead iodide (CsPbI_3_, noting that in our computations the bulk halide perovskite has a band gap lower than tetracene’s triplet energy).^18^ We find that the tetracene–CsPbI_3_ interface is not very energetically favorable to form, and when interfaces do form tetracene lies with its long axis perpendicular to the halide perovskite surface. We also present highly speculative calculations, suggesting that triplets remain strongly localized on tetracene, even at the interface. Our results go some way to explaining experimental observations, help rule out some posited reasons for the lack of triplet transfer, and suggest a clear route forward for future studies.

## 2 Brief Experimental Screening

We fabricated approximately 150 samples as low-band-gap (<1.25 eV) halide perovskite/singlet fission bilayers (see schematic, Supporting Information Figure 1a). A full list of all processing routes explored can be found in Supporting Information and experimental methods in SI B.

**Figure 1:**
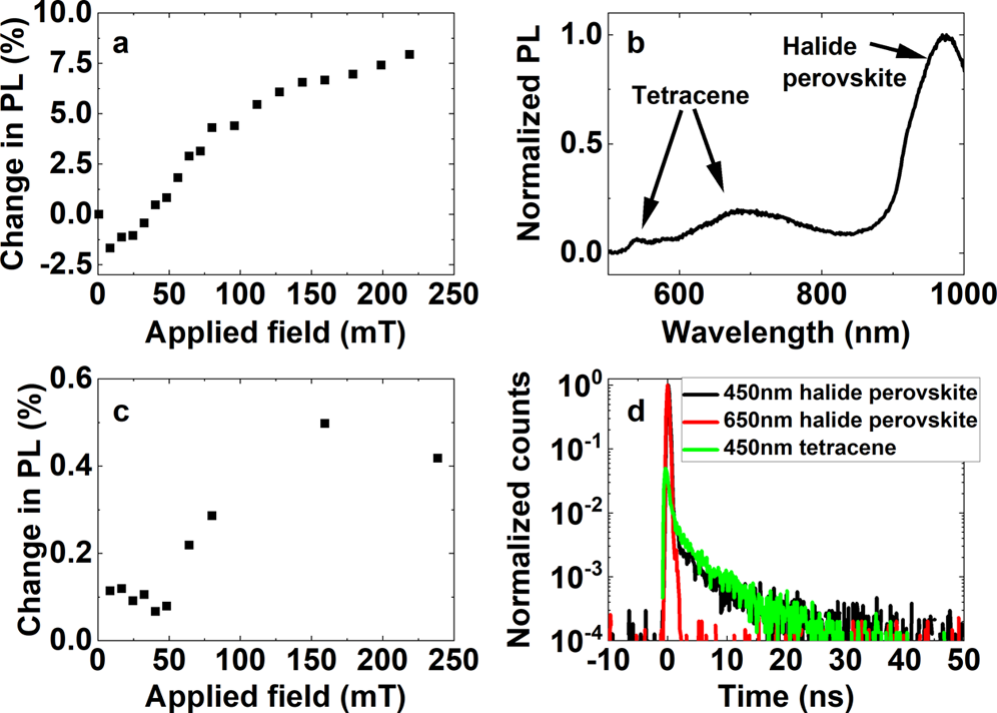
(a) Change in photoluminescence (PL) of tetracene to an applied magnetic field. The photoluminescence of a tetracene/FA_0.9_Cs_0.1_Pb_0.25_Sn_0.75_I_3_ bilayer under 405 nm illumination is presented in (b) (for tetracene evaporated on a spin-coated halide perovskite). This image was taken with an InGaAs camera using a 500 nm long-pass filter, so the tetracene signal (in the 550–700 nm region) appears weak due to poor camera response in this region. The plot is cut at 1000 nm due to the second-order signal occurring at longer wavelengths. The halide perovskite PL peaks at ∼950 nm. (c) Change in the halide perovskite’s photoluminescence under 405 nm illumination following application of a magnetic field (where a 900 nm long-pass filter is used so only the halide perovskite’s photoluminescence is observed). (d) Time-resolved photoluminescence of halide perovskite when exciting at 450 and 650 nm is presented, alongside suitably scaled TRPL from tetracene in the same bilayer under 450 nm illumination. The halide perovskite’s photoluminescence is short-lived due to hole transfer to tetracene.

We probed triplet transfer by applying a magnetic field to the singlet fission/halide perovskite bilayer and observing the change in the halide perovskite’s photoluminescence (PL) and via time-correlated single-photon counting (TCSPC). If there is net triplet transfer or dissociation at the interface, the halide perovskite’s PL will reduce at a high magnetic field as it will receive fewer triplets.^19^ Conversely, net singlet transfer will result in an increase in photoluminescence from the halide perovskite at high magnetic fields. We note that magnetic photoluminescence (which was carried out with continuous wave laser excitation) probes for both energy and charge transfer, indicating whether predominantly singlets or triplets are transferring, while TCSPC allows us to differentiate between energy and charge transfer (assuming that the lifetime of singlets or triplets is longer than the excited charge lifetime in the halide perovskite, as was the case in our measurements).

Two singlet fission materials were used in our screening experiments: tetracene and 1,6-diphenyl-1,3,5-hexatriene (DPH).^20−23^ The triplet energies in these molecules (∼1.3 and 1.5 eV, respectively) are larger than the selected halide perovskite’s band gap (∼1.25 eV), and they both have high triplet yields (with >50% of singlets converted to triplets).

When we began this study, it had recently been observed that adding small quantities of transition metals into halide perovskites could change their work function.^24^ A similar effect could be seen by changing the quantity of cesium at the A site of the halide perovskite.^25^ In both cases, the halide perovskite’s band gap remains constant. Halide perovskites with different work functions were fabricated according to these two methods. All samples had either 50:50 or 25:75 ratios of lead to tin, corresponding to band gaps in the 1.2–1.25 eV range. We fabricated halide perovskite films that were thin (50–200 nm) to increase any singlet fission contribution to the halide perovskite’s photoluminescence, and we also explored the effects of varying the singlet fission material thickness deposited (from well above to well below the absorption length of the material).

We present an example of the results obtained from these screening experiments in Figure 1, for a tetracene/FA_0.9_Cs_0.1_Pb_0.25_Sn_0.75_I_3_ bilayer (with tetracene evaporated on to a spin-coated halide perovskite, where FA is formamidinium). We present the magnetic PL response of evaporated tetracene in Figure 1a, showing an increase in PL at a high magnetic field, as expected. In Figure 1b, the photoluminescence of the bilayer is presented (from 500 to 1000 nm). Tetracene can be weakly observed in the 500–700 nm region (it is weak due to the camera used, see figure caption), and the halide perovskite PL peaks at ∼975 nm. We were able to selectively monitor the halide perovskite’s PL change with a magnetic field using a 900 nm long-pass filter, as shown in Figure 1c. At a low magnetic field, the change in PL is <0.2%, which is within the experimental noise. However, at a high magnetic field, the halide perovskite’s PL increases to a significant level (>0.4%), indicating net singlet transfer from tetracene. Singlet transfer is confirmed by TCSPC, as shown in Figure 1d. By exciting the bilayer above and below tetracene’s band gap and observing the halide perovskite’s time-resolved photoluminescence (TRPL), we observe that the longer-lived component of this TRPL coincides well with the TRPL from tetracene (in the bilayer). Furthermore, we find that the halide perovskite’s TRPL shows that nothing longer lived than the singlet transfer, suggesting that any triplet transfer from tetracene is negligible (noting triplets are typically longer lived than singlets).

In all cases explored, we observed singlet fission in the organic layer but no net triplet transfer to the halide perovskite (though singlet transfer was readily observed). To better understand organic–inorganic interfaces and potentially explore the reasons for the lack of triplet transfer from singlet fission materials, we modeled a proto-typical singlet fission–halide perovskite bilayer.

## 3 Modeling Background

It has recently become possible to study some simple interfaces with first-principles computational methods. For example, density functional theory (DFT) calculations (corroborated by X-ray diffraction experiments) have helped confirm the geometry of the interface between tetracene and silicon,^26,27^ where tetracene thin films were found to orient with their long axis perpendicular to the semiconductor surface.

Here, we model tetracene and CsPbI_3_ using DFT. We selected CsPbI_3_ for this study as lead–iodine-based perovskites are some of the best understood halide perovskites, the cation has spherical symmetry (unlike methylammonium or formamidinium) and an inorganic lead halide perovskite reduces the number of atomic species in the system, simplifying modeling while maintaining the same basic electronic structure (noting the electronic structure of halide perovskites is governed by the metal–halide framework). As several earlier studies have shown, it is difficult to obtain triplet transfer to several different inorganic semiconductors,^12,13^ we postulate that the reason for the lack of triplet transfer lies primarily within the singlet fission material. This is further supported by halide perovskites sensitizing triplet states in singlet fission materials.^28^ Therefore, our modeling focuses on correctly reproducing tetracene’s electronic states (at the expense of correctly modeling the halide perovskite’s electronic states).

We first discuss each system in isolation and quantify the effects of surface terminations and band gap corrections. We then present geometry arrangements and resulting electronic structures of a single tetracene molecule and bulk tetracene films on a halide perovskite surface. As in previous studies,^26^ we found that post-DFT *GW* and BSE calculations (required to calculate excitonic states) of full interfaces were not feasible. Instead, we present highly speculative toy models between tetracene and a halide perovskitelike structure. We focus on correctly reproducing tetracene’s electronic states at the interface (at the expense of the halide perovskite). We note that in all interface models we have assumed the best-case scenario of perfect surfaces, with no ion migration, surface defects, or other detrimental effects present, and that triplets can readily diffuse to the surface (all other effects would further limit triplet transfer). All computational details for subsequent sections can be found in Supporting Information C.

## 4 Tetracene

Tetracene is one of the most widely studied singlet fission materials.^8,20,29−31^ It undergoes singlet fission endothermically and, consequently, the process proceeds at a slower pace than in other molecules.^21^ Among singlet fission materials, its triplet energy is one of the highest, making it one of the most relevant for solar energy applications.^32^ Initially, we carried out geometry relaxations of bulk tetracene unit cells using several different exchange-correlation functionals and van der Waals corrections. We found that the PBE generalized gradient approximation, coupled to a Tkatchenko–Scheffler (TS) van der Waals semiempirical correction, best reproduces experimental tetracene lattice parameters, in agreement with other analyses^33,26^ (see Supporting Information Table I for calculations on tetracene, Al-Saidi and co-workers for an assessment of the TS correction on extended systems and Saidi and co-workers for a discussion of using PBE and a TS correction on halide perovskites^34,35^). We used this functional and van der Waals correction in all subsequent calculations.

**Figure 2:**
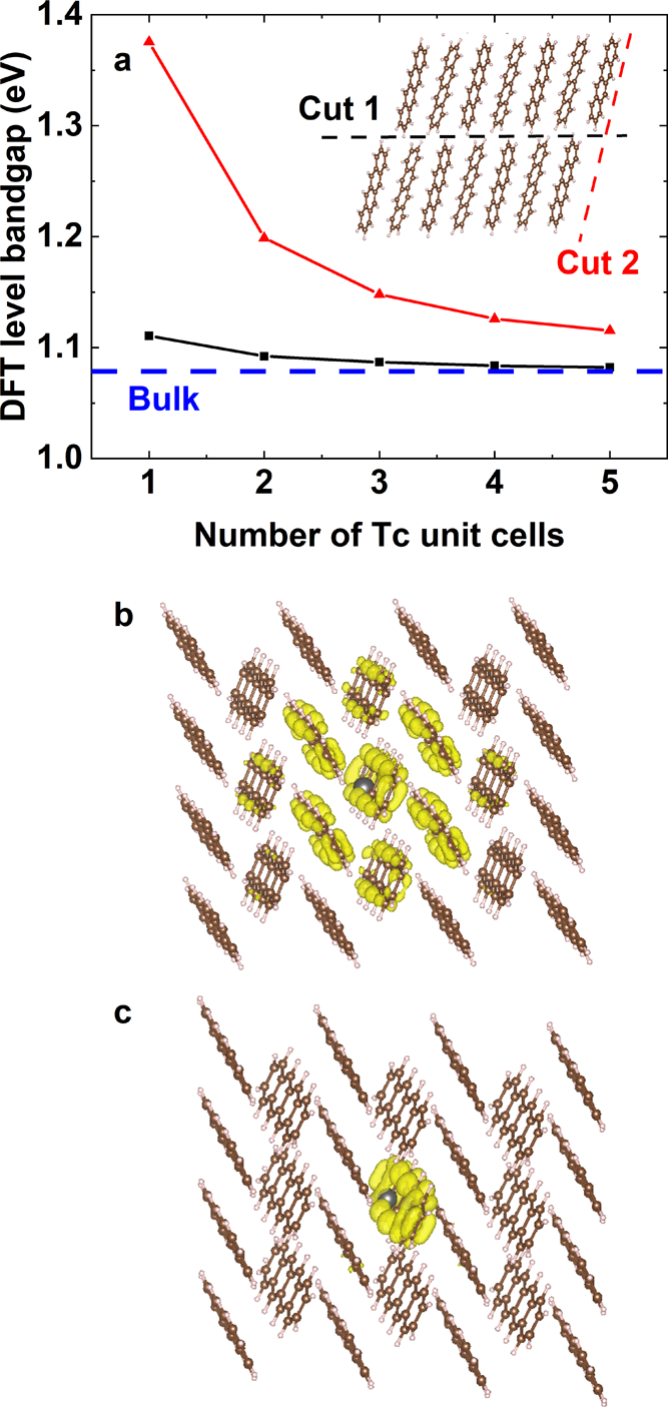
(a) DFT-level band gap of relaxed tetracene films with different surface terminations versus the number of tetracene molecules in the vacuum direction. The inset shows the two surface terminations considered and the dashed blue line the bulk result. The singlet and triplet electron charge densities for hole (gray sphere) fixed on a carbon atom are presented in (b) and (c), respectively.

In Figure 2a, we plot the DFT-level band gap for different relaxed tetracene surfaces versus the number of tetracene repeating units in the nonperiodic direction. Modeled surfaces are termed “cut 1” and “cut 2” (other cuts were not commensurate with the halide perovskite unit cell), as shown in the inset. While cut 1 has relatively little effect on the DFT-level band gap, cut 2 results in a band gap increase of ∼0.25 eV for a single repeating unit, which reduces to within 0.1 eV by three repeating units. This demonstrates that there is relatively little electronic interaction between tetracene layers (i.e., in the direction perpendicular to cut 1); rather, electronic states are mostly localized to one layer.

Tetracene’s (optically) excited states typically exist as singlets and triplets. We used a one-shot *G*_0_*W*_0_ calculation together with the Bethe–Salpeter equation to calculate the singlet and triplet states of bulk tetracene. The lowest-energy singlet and triplet states of relaxed tetracene have energies of 2.08 and 1.24 eV, respectively, which increase to 2.22 and 1.27 eV when using experimental lattice parameters, in good agreement with experimental results.^21,36^ While it is not possible to plot excitonic wave functions (as these are two-particle states requiring six spatial coordinates), it is possible to plot electron or hole charge densities with the other particle fixed in place. In Figure 2b,c, we plot the electronic charge density for the lowest-energy singlet and triplet states, respectively, with the hole fixed at a carbon atom (gray sphere). The singlet state is delocalized over several molecules, but the triplet is almost fully localized to a single molecule, as has been previously discussed by others.^37^ We found this to be the case for the hole being fixed at several different positions within the tetracene film. Furthermore, neither the singlet nor triplet states have significant wave function overlap between the planes of the film (in a direction perpendicular to cut 1), again showing that films have quasi-two-dimensional electronic states.

## 5 Cesium Lead Iodide

**Figure 3:**
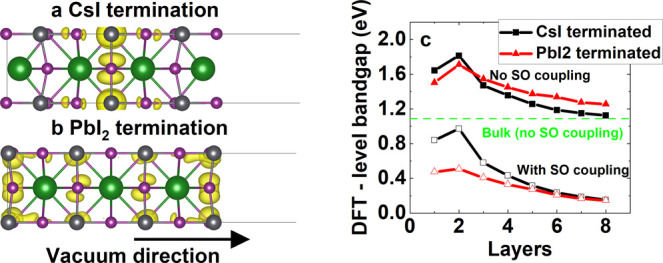
Panels (a, b) plot the valence band charge density (at the band edge) of CsI- and PbI_2_-terminated surfaces. In panel (c), the DFT-level band gap is presented for different numbers of repeating units in the vacuum direction, for (100) CsI- and PbI_2_-terminated surfaces, with and without spin–orbit (SO) coupling. The dashed line marks the bulk result in the case of no spin–orbit coupling. In panels (a, b), all calculations include spin–orbit coupling.

We discuss the electronic structure of bulk CsPbI_3_ in Supporting Information D. This section highlights that DFT-level band gap predictions (without spin–orbit, SO, coupling) are comparable to more complete calculations accounting for both SO coupling and *G*_0_*W*_0_ corrections, which is important in subsequent simulation results. When we consider an interface, there are many possible surface terminations for a cubic inorganic structure. First-principles calculations on related halide perovskites suggest that (100) surfaces are the most likely to form, so our study focuses on these surfaces.^38^ We consider two possible surface terminations of this plane: PbI_2_ and CsI (noting that these are neutrally charged surfaces so more stable than other surfaces, as has previously been discussed in other comparable systems^39^). In all our modeling, the same surface was used on both sides of the halide perovskite, mitigating surface dipole effects. We present the valence band charge densities for these two relaxed surface terminations in Figure 3a,b. While the CsI termination has the valence band confined to the bulk, PbI_2_ termination has a significant surface contribution (i.e., a dangling bond). This gives two significantly different situations, so our subsequent calculations explore both surfaces. We note that PbI_2_ surfaces are lower in energy so are more likely to form in reality. In both materials, the conduction band is confined to the bulk.

The electronic structure of halide perovskites is fundamentally three-dimensional, so it is strongly affected by quantum confinement. In Figure 3c, we present the DFT-level band gap of CsPbI_3_ for both relaxed surface terminations, as a function of the number of repeating units in the vacuum direction, both with and without spin–orbit coupling. In all cases, a significant increase in band gaps is found when compared to the bulk, as expected. We find that CsI-terminated surfaces have slightly larger band gaps, which is attributed to more confined states (c.f. Figure 3a,b).

## 6 Tetracene Molecules on Halide Perovskite Surfaces

**Figure 4:**
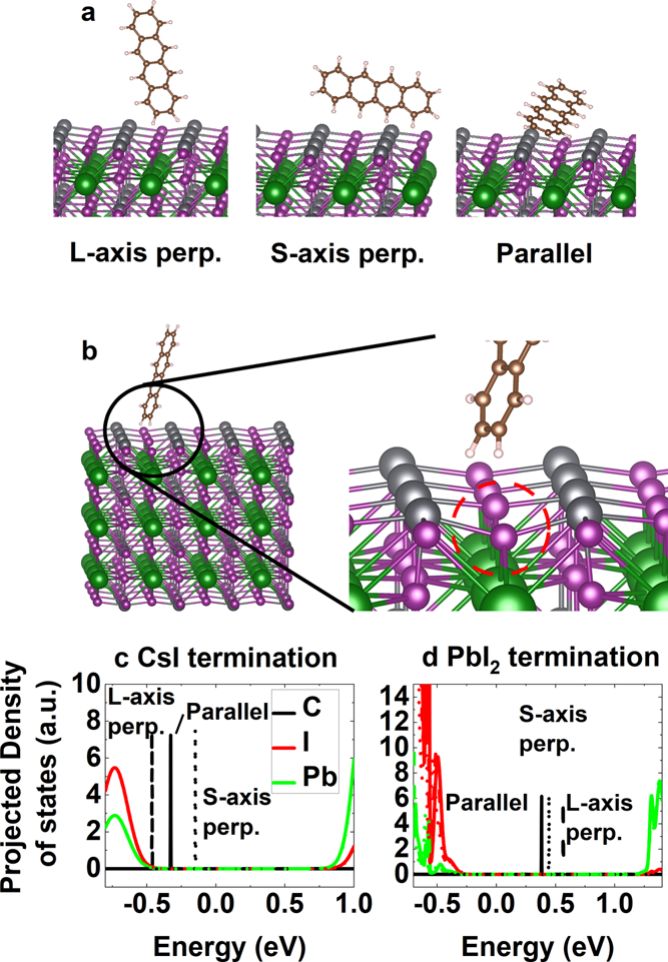
Three possible tetracene orientations on the halide perovskite surface are presented in (a). An example of a relaxed geometry is shown in (b), for PbI_2_ surface termination with tetracene’s long axis perpendicular to the surface. The region of interaction is enlarged, showing the halide perovskite surface distortion due to the tetracene. Panels (c, d) show the projected density of states, in arbitrary units, without spin–orbit coupling, for the three tetracene orientations for CsI- and PbI_2_-terminated surfaces, respectively. Here, parallel is shown with a solid line, the short-axis perpendicular (S-axis perp.) with a short dashed line, and the long-axis perpendicular (L-axis perp.) with a long dashed line. Energy has been shifted arbitrarily to align the halide perovskite valence bands, i.e., 0 energy is an arbitrary value. In all cases, the carbon states are the highest occupied states and represent the valence band for this situation.

To simulate a halide perovskite thin film, we modeled three halide perovskite layers in the nonperiodic direction, as interatomic distances in the center of this structure were comparable to distances in bulk CsPbI_3_ (see Supporting Information Table II) and computation was not too expensive. As has been discussed elsewhere,^26,27,40^ there are three main orientations for a tetracene molecule to lie on a halide perovskite surface: with the long axis of the molecule perpendicular; the short axis of the molecule perpendicular; or face-on to the halide perovskite (termed parallel, see Figure 4a).

We carried out geometry relaxations for the three tetracene orientations on both halide perovskite surfaces (i.e., six simulations). The relaxed energy is relatively independent of where the tetracene is initially placed above a semiconductor,^26^ so we only considered one starting position for each geometry relaxation. We present an example of a relaxed geometry in Figure 4b. The tetracene molecule is observed to remain unchanged, while the halide perovskite surface distorts. We found qualitatively similar results in all simulations. This is reasonable as halide perovskites have surprisingly low Young’s moduli for inorganic semiconductors, comparable to those of relevant organic crystals.^41−44^

The formation energy between the halide perovskite surface and the tetracene molecule is given by *E*_formation_ = *E*_interface_ – (*E*_perov,slab_ + *E*_tetracene,molecule_), that is, the energy of the interface less the energy of the halide perovskite slab and the tetracene molecule in vacuum. We present *E*_formation_ for the six geometries considered in Table 1. Results suggest that the parallel tetracene arrangement has the most favorable formation energy (noting in other simulations, the geometry relaxation found a local energy minimum). This is in agreement with other examples of rodlike molecules on the surface of inorganic semiconductors.^26,27,40^ Furthermore, in all cases, we find that the interaction energy is observed to be weak: for comparison, the formation energy for a tetracene film is ∼−2 eV per molecule.

**Table 1:** Formation Energy, *E*_formation_, for Three Orientations of a Tetracene Molecule on CsI- and PbI_2_-Terminated Halide Perovskite Surfaces

tetracene orientation	*E*_formation_ (eV)
CsI Termination	
long-axis perpendicular	–0.50
short-axis perpendicular	–0.26
parallel	–0.69
PbI_2_ Termination	
long-axis perpendicular	–1.03
short-axis perpendicular	–0.82
parallel	–1.25

To understand band alignment at each interface, we calculated the projected density of states (PDOS), with projection onto each species’ atomic orbitals. We neglected spin–orbit coupling as this gives a similar halide perovskite band gap to that obtained with spin–orbit coupling and a *G*_0_*W*_0_ correction at significantly less computational cost (see Supporting Information D). Our results are presented in Figure 4c,d. For CsI-terminated surfaces, as the halide perovskite’s valence band is localized in the bulk of the structure (c.f. Figure 3a), its PDOS is unchanged for different tetracene orientations (Figure 4c). For the (energetically favored) parallel and short-axis perpendicular orientations, tetracene’s valence band lies above that of the halide perovskite, while for the long-axis perpendicular model, its valence band aligns with the halide perovskite’s valence band maximum. For PbI_2_ termination, as the halide perovskite’s valence band has some surface character, we find that there is a small variation in the halide perovskite’s PDOS for the different simulations (Figure 4d). Importantly, tetracene’s valence band is above the halide perovskite’s in all cases and significantly more offset from the halide perovskite’s valence band than in the CsI-terminated case. We attribute this to PbI_2_-terminated surfaces being lower in energy than CsI-terminated surfaces rather than a significant change in the tetracene’s electronic structure. Tetracene’s valence band being above the halide perovskite is indicative of tetracene being a good hole extracting material, as has been observed experimentally in halide perovskite–tetracene interfaces.^45^

## 7 Tetracene–Halide Perovskite Thin-Film Interface

To model interfaces between bulk materials, unit cells with commensurate in-plane lattice parameters need to be found. We identified two tetracene–halide perovskite interfaces with tetracene’s cut 1 surface termination and one with cut 2 surface termination. They are shown in Supporting Information Figure S3. We were not able to find any commensurate unit cells of reasonable size for tetracene’s third possible exposed plane. These three simulations are termed “cut 1 no rotation,” “cut 1 with rotation” and “cut 2.” The two cut 1 simulations have normal strains of <3% and shear strain of ∼10%, while cut 2 has both normal and shear strains on the order of 5% (see Supporting Information Table III for all strains). Two tetracene unit cells were used in the nonperiodic direction for cut 1 models and four tetracene units cells for cut 2 (based on results shown in Figure 2a). This resulted in large cells for DFT simulations so no vacuum layer was used, i.e., there were two interfaces between tetracene and the halide perovskite. This also reduced the possibility of any dipole effects. The distance between adjacent halide perovskite and adjacent tetracene layers was at least as large as the vacuum spacing required to prevent interaction between adjacent layers of the same material in the nonperiodic direction. As tetracene and halide perovskites have comparable Young’s moduli, we allowed lattice parameters to vary in geometry relaxations.^41,42^

**Figure 5:**
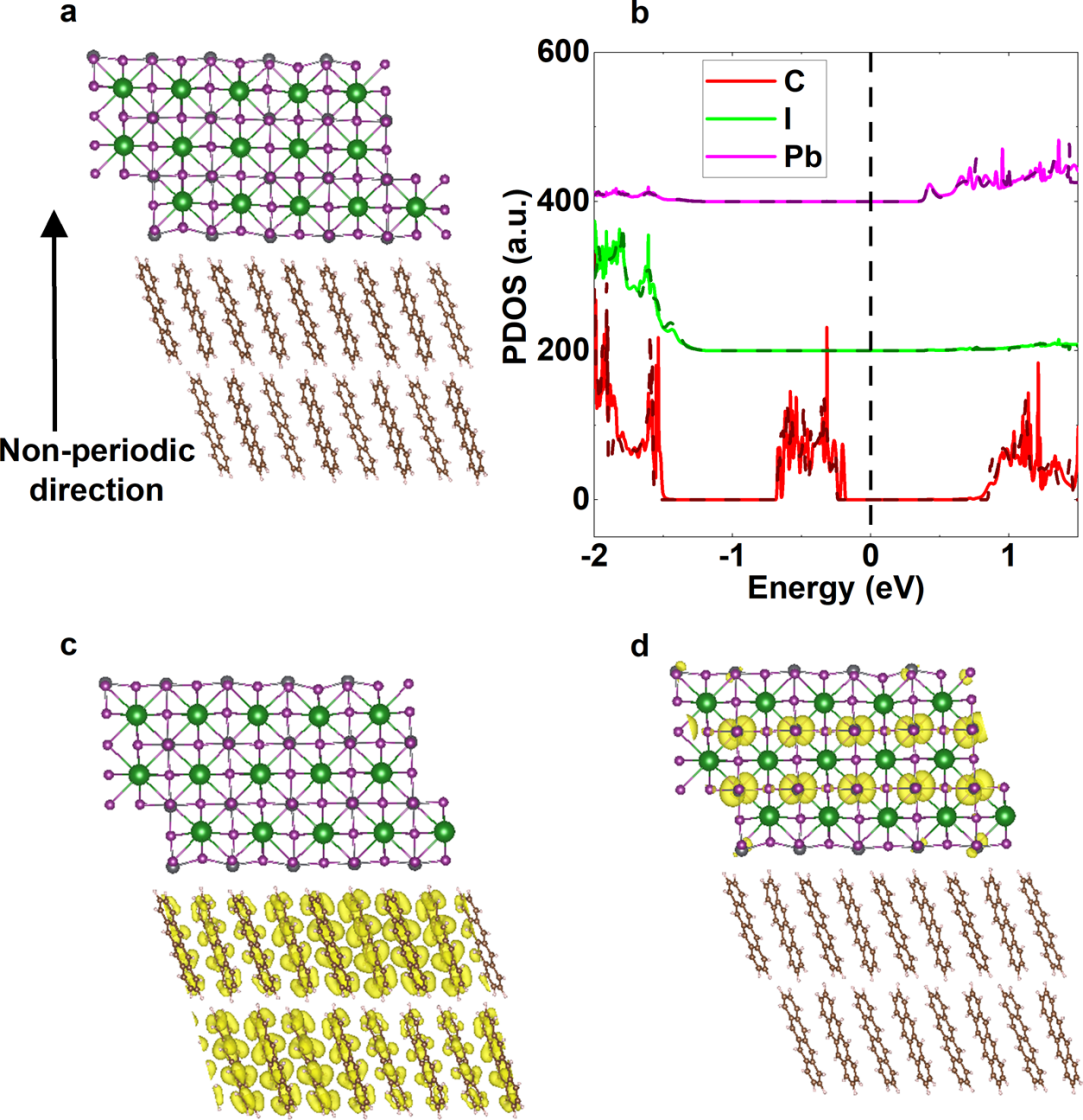
(a) Fully relaxed geometry of cut 1 no rotation with PbI_2_ surface termination. The corresponding projected density of states (PDOS) without spin–orbit coupling is shown in (b). Here, the dashed vertical black line marks the Fermi level, and the dashed colored lines mark the PDOS for isolated relaxed PbI_2_ and tetracene slabs in vacuum. The valence and conduction band charge densities of this interface at band edges are presented in (c) and (d).

We ran geometry relaxations for all simulation cells. All cuts ran successfully except cut 2 with CsI termination, where the halide perovskite structure fell apart during computation. We attribute this to the unit cell being too strained for the optimization to complete, and this geometry is not discussed further. We present a relaxed geometry in Figure 5a for PbI_2_-terminated cut 1 no rotation. In all cut 1 configurations, including that presented here, we found tetracene molecules to be almost entirely unchanged in position from a thin film of tetracene. For cut 2, we found that the spacing between tetracene molecules reduced slightly (<2% change) in the nonperiodic direction, especially for molecules adjacent to the interface. In all cases, the halide perovskite surface distorted more significantly.

We define the interface formation energy per tetracene molecule as
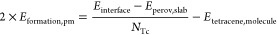
1where *N*_Tc_ is the number of tetracene molecules in the interface simulation. The factor of two arises from the double interface. We present formation energies in Table 2. Larger energy reductions are observed for CsI termination, which is explained by noting that bare CsI surfaces are less stable (higher in energy) than the PbI_2_ surfaces, so bulk films provide more significant stabilization. In both cases, the two cut 1 geometries have almost identical energies, suggesting that tetracene’s orientation on the halide perovskite surface is only weakly dependent on the halide perovskite. This is indicative of weak interaction between the two materials. For PbI_2_ termination, we find that cut 1 orientations are more favorable than cut 2, as is again expected for rodlike molecules.^40^ This predicted tetracene orientation should now be verified experimentally. All formation energies are significantly higher than for bulk tetracene (∼−2 eV per molecule), which suggests that tetracene will not bind strongly to the halide perovskite surface. It may instead form pillars or other structures with only small contact regions with the halide perovskite, as seen in our Experimental Section (c.f. Supporting Information Figure 1b) and has been discussed by others.^40^

**Table 2:** Formation Energies per Molecule, *E*_formation,pm_, for Relaxed Thin-Film Interfaces

tetracene orientation	*E*_formation,pm_ (eV)
CsI Termination	
cut 1 no rotation	–1.42
cut 1 with rotation	–1.41
PbI_2_ Termination	
cut 1 no rotation	–0.95
cut 1 with rotation	–0.95
cut 2	–0.92

We show the projected density of states for PbI_2_-terminated cut 1 no rotation in Figure 5b (PDOS for other cut 1 simulations are presented in Supporting Information Figure S4). As in the case of a single molecule, the tetracene’s valence band is located above the halide perovskite’s. This was the case for all other simulations. Overlaid on this plot in the dashed lines are the PDOS for halide perovskite and tetracene slabs (independently) isolated in vacuum. For the halide perovskite, the isolated PDOS overlaps almost perfectly with the valence and conduction bands. We find that tetracene’s PDOS at the interface is slightly broadened with respect to isolated tetracene, which we attribute to each tetracene molecule being in a slightly different electronic environment at the interface. This PDOS demonstrates that there is little interaction between the halide perovskite and tetracene, as their states can be well reproduced by isolated slabs.

We present the valence and conduction band charge densities (at the band edge) for cut 1 no rotation, for PbI_2_ termination, in Figure 5c,d, respectively. Both charge densities are isolated to one material only (tetracene and halide perovskite, respectively), again implying that there is little change in electronic states at the interface. When looking further into the valence band (at the k-point corresponding to the valence band maximum), no states are found to have significant charge density in both the halide perovskite and the tetracene.

## 8 Toy Interface with Exciton Visualization

To model triplet excitons, it is necessary to solve the Bethe–Salpeter equation. We attempted this on the full interfaces presented in the previous section but found it was not computationally feasible. A similar limitation was recently highlighted in Janke and co-workers’ study of pentacene and tetracene on passivated silicon surfaces.^26^ Other studies have focused on the development of fragment-based (post-DFT) *GW* and BSE calculations, allowing for the modeling of interfaces.^46^ However, in fragment-based approaches, the exchange interaction needs to be neglected, meaning singlet and triplet states cannot be differentiated.

**Figure 6:**
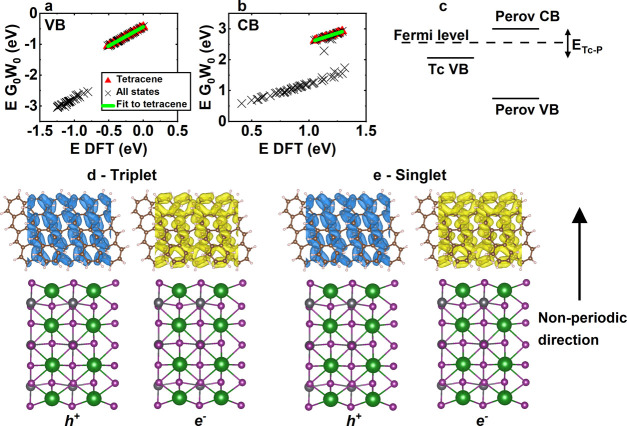
DFT energies versus *G*_0_*W*_0_ energies for the CsI-terminated toy model’s valence band (VB) and conduction band (CB), with spin–orbit coupling, are shown in (a) and (b). The legend in (a) applies to (a) and (b). The schematic in (c) shows relative band energies at the interface, with *E*_Tc–P_ being the difference in energy between the tetracene’s valence band and the halide perovskite’s conduction band. Average hole (h^+^) and electron (e^–^) charge densities for the lowest-energy triplet and singlet excitons for the CsI-terminated surface are plotted in (d) and (e), for the *G*_0_*W*_0_ correction shown in (a) and (b) applied. Isosurfaces show the 95% probability boundary for all plots (i.e., there is a 95% probability that the electron–hole will be found inside the plotted surfaces).

Despite these limitations, we decided to carry out highly speculative calculations to explore what excitonic properties can be modeled at an interface with modern computational methods and resources. We note that it is unclear whether full excitonic states will form at the interface or whether charges dissociate prior to reaching the interface, but here we aim to increase understanding of the possible states at the interface and ascertain what current computational methods reveal. To this end, we constructed small toy interfaces consisting of a single tetracene unit cell and a single (in plane) CsPbI_3_ unit cell. In these models, we oriented tetracene perpendicular to the interface (cut 1 orientation, as this was the most energetically favorable) and the halide perovskite again had three repeating units in the nonperiodic direction. Unfortunately, simulation cells with cut 2 orientations were too computationally intensive to complete (due to the larger unit cell size). As we are interested primarily in tetracene’s electronic states, the model’s in-plane lattice parameters were constrained to those of tetracene. To achieve this, one of the halide perovskite’s in-plane lattice parameters was increased by ∼20% and the other reduced by ∼3%. Again we modeled both CsI and PbI_2_ terminations. We carried out geometry optimizations (with in-plane lattice parameters constrained) without any vacuum spacing. However, for *G*_0_*W*_0_ and BSE calculations, the lack of vacuum spacing resulted in the interaction between adjacent unit cells in the nonperiodic direction. Therefore, we introduced a vacuum layer (the same size as the “filled” unit cell) and used a Coulomb cutoff to prevent long-range interactions between repeating unit cells. An example of the unit cell studied can be seen in Figure 6d.

We calculated the PDOS of these small models (with vacuum spacing), and our results are presented in Supporting Information Figure S5a,b. Electronic states were positioned similarly to those presented in previous sections, with the same atomic orbitals contributing to the halide perovskite’s valence and conduction bands, and tetracene’s valence band being above that of the halide perovskite. Importantly, we found that the halide perovskite’s band gap had increased, due to the larger in-plane lattice parameter increasing quantum confinement. Density of state calculations including spin–orbit coupling, plotted in Supporting Information Figure S5c,d, demonstrate that for PbI_2_ termination there is no band gap in this system, preventing post-DFT calculations with spin–orbit coupling on this system.

We were able to carry out *G*_0_*W*_0_ and BSE calculations on these toy interface models. However, further simplifications were needed for calculations to proceed: we neglected the nonlocal commutator and we reduced the maximum reciprocal lattice vector size with respect to DFT calculations. The latter approximation is equivalent to reducing the cutoff energy in a DFT calculation but was found to have only small effect (<0.01 eV) due to smaller reciprocal lattice grids being required in *G*_0_*W*_0_ calculations (see Supporting Information C). We found that both approximations were reasonable by carrying out one calculation without these approximations—minimal difference in results was observed.

*G*_0_*W*_0_ calculations on the toy interfaces, alongside on just tetracene and toy halide perovskite, allow for an estimation of the changes that would occur to the PDOS calculations already presented. Specifically, we present *G*_0_*W*_0_ corrections in Supporting Information Table IV, focusing on the change in energy offset between halide perovskite and tetracene valence bands. In general, our results point toward the difference in energy between the halide perovskite and tetracene valence bands being larger than what was calculated at DFT level by ∼ 1 eV. As is discussed in Supporting Information E, this would be partly offset by calculations including spin–orbit coupling in the halide perovskite (reducing energy difference between the halide perovskite and tetracene valence bands), so we consider that the PDOS presented above gives the correct qualitative conclusions.

We plot DFT energies versus *G*_0_*W*_0_ energies for valence and conduction bands in Figure 6a,b for the CsI-terminated toy model with spin–orbit coupling. Unlike in most situations, corrections to DFT-level energies do not form straight lines. This is because the halide perovskite and tetracene energy corrections are significantly different in magnitude (as tetracene’s electronic states are more localized). Furthermore, there are some mixed states including both parts of the tetracene and halide perovskite wave function, which carry intermediate energy corrections. To model tetracene’s electronic states correctly at the interfaces, we fit a straight line to states where the DFT wave function is fully localized on tetracene, as shown in the figure. These fits allow for a comparison of the tetracene’s *G*_0_*W*_0_ level band gap with that of bulk tetracene and isolated tetracene sheets (cut 1). We present results in Supporting Information Table V. The tetracene’s *G*_0_*W*_0_ level band gap is lowered at all interfaces, which is attributed to the halide perovskite stabilizing single-particle excited states in tetracene.

We show a schematic of energy levels in the toy model interfaces in Figure 6c. The only states close to the Fermi level are tetracene’s valence band and the halide perovskite’s conduction band. We define the energy difference between these states as *E*_Tc–P_. This means that we can explore exciton dissociation at the interface, but not full exciton transfer. We note that we are therefore exploring a different situation to the reverse of triplets in organic molecules being sensitized by halide perovskites, as has recently been observed experimentally.^47^

To carry out BSE (exciton) calculations, we applied scissor corrections to DFT energy levels. Scissor corrections shift DFT-level valence and conduction band states by constant gradients, as in the green lines in Figure 6a,b and add a constant value to the DFT-level band gap. Initially, we used the scissor corrections found from *G*_0_*W*_0_ fits, i.e., correctly replicating tetracene’s electronic states at the interface (as in Figure 6a,b). With these scissor corrections, for both CsI- and PbI_2_-terminated surfaces, we found tetracene’s triplet state to be the lowest-energy state at the interface. In calculations including spin–orbit coupling, we identified “triplet” and “singlet” states as triply/singly degenerate dark/bright states with significant electron and hole contributions on tetracene. We note that both the oscillator strength and the shape of the triplet/singlet wave functions within spin–orbit coupling are fully comparable to without spin–orbit coupling. At both interfaces, we found the singlet and triplet energies to be extremely comparable to those of a tetracene cut 1 layer in a vacuum (see Supporting Information Table VI). For example, for CsI termination with spin–orbit coupling, we found the triplet states at 1.17 eV, while for the same tetracene geometry in a vacuum (i.e., no halide perovskite present), a triplet energy of 1.11 eV was calculated, in agreement within calculation error. This is indicative of tetracene’s excitonic states being relatively unaffected by the presence of the halide perovskite.

We plot the average hole and electron positions of the lowest-energy triplet and singlet states for CsI termination in Figure 6d,e, respectively (with scissor correction applied, which correctly reproduces tetracene’s electronic states). Both states are found to be strongly localized to tetracene. Specifically, for both triplet and singlet, there is less than a 3% probability of the electron or hole being within the halide perovskite. Importantly, there are lower-energy charge-transfer states than the singlet state in this model where the electron is fully localized on the halide perovskite. We present equivalent results for PbI_2_-terminated surfaces in Supporting Information F. Again we calculate well-formed singlet and triplet states localized on tetracene only. We note that the lack of hybridization between tetracene and halide perovskite excitonic states does not definitively rule out the possibility of charge transfer, especially when considering wave function overlap, but further calculations of wave function overlap and transfer probabilities were found not to be computationally feasible.

Figure 6 is in the case of *E*_Tc–P_ being larger than tetracene’s triplet energy. To explore triplet states in the presence of lower-energy states, we then slightly altered the scissor correction, reducing *E*_Tc–P_ and making charge-transfer states (with the electron localized on the halide perovskite and the hole on the tetracene) lower in energy than tetracene’s triplet energy (see Supporting Information Table V). This was to explore whether singlet/triplet states would readily hybridize with other states even when any energy barrier to this is removed. We obtained almost identical results with singlet and triplet states remaining strongly localized on tetracene at the interface. Furthermore, for charge-transfer states, the electron–hole remained fully localized on the halide perovskite/tetracene. Our results therefore suggest that one possible issue for exciton dissociation is the spatial separation of relevant states.

Changing the scissor correction means tetracene’s electronic states are no longer exactly reproduced fully at the interface. As a final confirmation to show that results with altered scissor corrections are valid, we replaced lead with tin to form an SnI_2_-terminated surface. Following geometry relaxations, we carried out the same *G*_0_*W*_0_ and BSE calculations. We obtained charge-transfer states lower in energy than tetracene’s triplet energy for the physically correct scissor shift. Importantly, our results were found to be similar to those for the PbI_2_ discussed in Supporting Information F, with triplet states remaining localized on tetracene. This confirms that results calculated with approximate scissor corrections are valid.

We attempted to extend these calculations by further altering the scissor correction, reducing the halide perovskite’s band gap to less than tetracene’s triplet energy. This required extreme scissor corrections and only resulted in charge-transfer states (with electron localized on the halide perovskite only). No conclusions can be drawn from this as nothing resembling a singlet or triplet state was calculated. We suggest that the extreme scissor correction leads to tetracene’s electronic states being reproduced extremely poorly.

In summary, we find that tetracene’s triplet and singlet states are strongly localized to tetracene at all interfaces considered. Furthermore, singlet and triplet energies are comparable to those for tetracene isolated in vacuum for all interfaces. These combined results suggest that for the optimal orientation of tetracene on the halide perovskite, tetracene’s excitonic states still exist at the interface and are relatively unaffected by the presence of an inorganic semiconductor.

## 9 Conclusions

We have presented a study of the interface between singlet fission materials and halide perovskites. This study was motivated by ∼150 experiments screening for triplet transfer from a singlet fission material to a halide perovskite. In our model, we found that tetracene behaved in a broadly similar way to other organic–inorganic interfaces, where the organic is a rodlike structure: lone organic molecules orient parallel to the halide perovskite surface, while films orient with the long axis perpendicular to the surface. In all cases, we found interface formation energies were much less favorable than for bulk tetracene and tetracene’s valence band was higher energy than the halide perovskite’s valence band, suggestive of tetracene being a good hole transporter. In general, we observed only weak electronic interaction between these two materials. Using small models, we were able to calculate the excitonic states between tetracene and a toy halide perovskitelike structure. We found that singlets and triplets remain localized on tetracene molecules at the interface, and tetracene’s excitonic energies were unaffected by the presence of the halide perovskite. Our results are indicative of only weak interaction between tetracene’s excitonic states and an inorganic semiconductor (for optimal molecular arrangements at the interface). We suggest that future work should focus on increasing electronic interaction at the interface by further exploring a chemical bonding between the two materials, improving interface formation energies (potentially exploring other halide perovskite-based materials^48^) and increasing the probability of triplet transfer. Our work lays the ground for achieving triplet transfer from a singlet fission material to a halide perovskite.

## Supporting Information Available

A: List of experiments undertaken screening for triplet transfer; B: list of other experimental methods; C: computational details; D: additional information on DFT calculations of bulk CsPbI_3_; E: additional information on *G*_0_*W*_0_ calculations for tetracene–halide perovskite interfaces; F: *G*_0_*W*_0_ calculations for PbI_2_-terminated surfaces; and G: supporting figures (PDF)

## References

[ref1] Yadav P.; Pandey K.; Bhatt V.; Kumar M.; Kim J. Critical aspects of impedance spectroscopy in silicon solar cell characterization: A review. Renewable Sustainable Energy Rev. 2017, 76, 1562–1578. 10.1016/j.rser.2016.11.205.

[ref2] Martha S. K.; Markevich E.; Burgel V.; Salitra G.; Zinigrad E.; Markovsky B.; Sclar H.; Pramovich Z.; Heik O.; Aurbach D.; Exnar I.; Buqa H.; Drezen T.; Semrau G.; Schmidt M.; Kovacheva D.; Saliyski N. A short review on surface chemical aspects of Li batteries: A key for a good performance. J. Power Sources 2009, 189, 288–296. 10.1016/j.jpowsour.2008.09.084.

[ref3] Yang Z.; Dou J.; Wang M. Interface Engineering in n-i-p Metal Halide Perovskite Solar Cells. Sol. RRL 2018, 2, 1800177 10.1002/solr.201800177.

[ref4] Andaji-Garmaroudi Z.; Abdi-Jalebi M.; Kosasih F. U.; Doherty T.; Macpherson S.; Bowman A. R.; Man G. J.; Cappel U. B.; Rensmo H.; Ducati C.; Friend R. H.; Stranks S. D. Elucidating and Mitigating Degradation Processes in Perovskite Light-Emitting Diodes. Adv. Energy Mater. 2020, 10, 2002676 10.1002/aenm.202002676.

[ref5] Stadlober B.; Haas U.; Maresch H.; Haase A. Growth model of pentacene on inorganic and organic dielectrics based on scaling and rate-equation theory. Phys. Rev. B 2006, 74, 165302 10.1103/PhysRevB.74.165302.

[ref6] DeQuilettes D. W.; Koch S.; Burke S.; Paranji R. K.; Shropshire A. J.; Ziffer M. E.; Ginger D. S. Photoluminescence Lifetimes Exceeding 8 s and Quantum Yields Exceeding 30% in Hybrid Perovskite Thin Films by Ligand Passivation. ACS Energy Lett. 2016, 1, 438–444. 10.1021/acsenergylett.6b00236.

[ref7] Zhang Y.; Liu M.; Eperon G. E.; Leijtens T. C.; McMeekin D.; Saliba M.; Zhang W.; de Bastiani M.; Petrozza A.; Herz L. M.; Johnston M. B.; Lin H.; Snaith H. J. Charge selective contacts, mobile ions and anomalous hysteresis in organic-inorganic perovskite solar cells. Mater. Horiz. 2015, 2, 315–322. 10.1039/C4MH00238E.

[ref8] Smith M. B.; Michl J. Singlet Fission. Chem. Rev. 2010, 110, 6891–6936. 10.1021/cr1002613. 21053979

[ref9] Tayebjee M. J. Y.; McCamey D. R.; Schmidt T. W. Beyond Shockley-Queisser: Molecular Approaches to High-Efficiency Photovoltaics. J. Phys. Chem. Lett. 2015, 6, 2367–2378. 10.1021/acs.jpclett.5b00716. 26266619

[ref10] Scholes G. D. Long-Range Resonance Energy Transfer in Molecular Systems. Annu. Rev. Phys. Chem. 2003, 54, 57–87. 10.1146/annurev.physchem.54.011002.103746. 12471171

[ref11] Dexter D. L. A Theory of Sensitized Luminescence in Solids. J. Chem. Phys. 1953, 21, 836 10.1063/1.1699044.

[ref12] Piland G. B.; Burdett J. J.; Hung T. Y.; Chen P. H.; Lin C. F.; Chiu T. L.; Lee J. H.; Bardeen C. J. Dynamics of molecular excitons near a semiconductor surface studied by fluorescence quenching of polycrystalline tetracene on silicon. Chem. Phys. Lett. 2014, 601, 33–38. 10.1016/j.cplett.2014.03.075.

[ref13] Macqueen R. W.; Liebhaber M.; Niederhausen J.; Mews M.; Gersmann C.; Jäckle S.; Jäger K.; Tayebjee M. J.; Schmidt T. W.; Rech B.; Lips K. Crystalline silicon solar cells with tetracene interlayers: The path to silicon-singlet fission heterojunction devices. Mater. Horiz. 2018, 5, 1065–1075. 10.1039/C8MH00853A.

[ref14] Tabachnyk M.; Ehrler B.; Gélinas S.; Böhm M. L.; Walker B. J.; Musselman K. P.; Greenham N. C.; Friend R. H.; Rao A. Resonant energy transfer of triplet excitons from pentacene to PbSe nanocrystals. Nat. Mater. 2014, 13, 1033–1038. 10.1038/nmat4093. 25282509

[ref15] Thompson N. J.; Wilson M. W. B.; Congreve D. N.; Brown P. R.; Scherer J. M.; Bischof T. S.; Wu M.; Geva N.; Welborn M.; Voorhis T. V.; Bulović V.; Bawendi M. G.; Baldo M. A. Energy harvesting of non-emissive triplet excitons in tetracene by emissive PbS nanocrystals. Nat. Mater. 2014, 13, 1039–1043. 10.1038/nmat4097. 25282507

[ref16] Einzinger M.; Wu T.; Kompalla J. F.; Smith H. L.; Perkinson C. F.; Nienhaus L.; Wieghold S.; Congreve D. N.; Kahn A.; Bawendi M. G.; Baldo M. A. Sensitization of silicon by singlet exciton fission in tetracene. Nature 2019, 571, 90–94. 10.1038/s41586-019-1339-4. 31270480

[ref17] Huang T.; Koh T. T.; Schwan J.; Tran T. T.-T.; Xia P.; Wan K.; Mangolini L.; Tang M. L.; Roberts S. T. Bidirectional triplet exciton transfer between silicon nanocrystals and perylene. Chem. Sci. 2021, 12, 6737–6746. 10.1039/D1SC00311A. 34040750 PMC8132999

[ref18] Green M. A.; Ho-Baillie A.; Snaith H. J. The emergence of perovskite solar cells. Nat. Photon. 2014, 8, 506–514. 10.1038/nphoton.2014.134.

[ref19] Merrifield R. E. (Triplet Annihilation, Simple) Theory of Magnetic Field Effects on the Mutual Annihilation of Triplet Excitons. J. Chem. Phys. 1968, 48, 4318 10.1063/1.1669777.

[ref20] Merrifield R.; Avakian P.; Groff R. Fission of singlet excitons into pairs of triplet excitons in tetracene crystals. Chem. Phys. Lett. 1969, 3, 386–388. 10.1016/0009-2614(69)80144-2.

[ref21] Wilson M. W. B.; Rao A.; Johnson K.; Gélinas S.; Di Pietro R.; Clark J.; Friend R. H. Temperature-independent singlet exciton fission in tetracene. J. Am. Chem. Soc. 2013, 135, 16680–16688. 10.1021/ja408854u. 24148017

[ref22] Dillon R. J.; Piland G. B.; Bardeen C. J. Different rates of singlet fission in monoclinic versus orthorhombic crystal forms of diphenylhexatriene. J. Am. Chem. Soc. 2013, 135, 17278–17281. 10.1021/ja409266s. 24171495

[ref23] Wakasa M.; Kaise M.; Yago T.; Katoh R.; Wakikawa Y.; Ikoma T. What Can Be Learned from Magnetic Field Effects on Singlet Fission: Role of Exchange Interaction in Excited Triplet Pairs. J. Phys. Chem. C 2015, 119, 25840–25844. 10.1021/acs.jpcc.5b10176.

[ref24] Klug M. T.; Osherov A.; Haghighirad A. A.; Stranks S. D.; Brown P. R.; Bai S.; Wang J. T.-W.; Dang X.; Bulović V.; Snaith H. J.; Belcher A. M. Tailoring metal halide perovskites through metal substitution: influence on photovoltaic and material properties. Energy Environ. Sci. 2017, 10, 236–246. 10.1039/C6EE03201J.

[ref25] Prasanna R.; Gold-Parker A.; Leijtens T.; Conings B.; Babayigit A.; Boyen H. G.; Toney M. F.; McGehee M. D. Band Gap Tuning via Lattice Contraction and Octahedral Tilting in Perovskite Materials for Photovoltaics. J. Am. Chem. Soc. 2017, 139, 11117–11124. 10.1021/jacs.7b04981. 28704048

[ref26] Janke S. M.; Rossi M.; Levchenko S. V.; Kokott S.; Scheffler M.; Blum V. Pentacene and Tetracene Molecules and Films on H/Si(111): Level Alignment from Hybrid Density Functional Theory. Electron. Struct. 2020, 2, 035002 10.1088/2516-1075/ab9bb5.

[ref27] Niederhausen J.; Aldahhak H.; MacQueen R. W.; Schmidt W. G.; Gerstmann U.; Lips K. Tetracene ultrathin film growth on silicon. Langmuir 2020, 36, 9099–9113. 10.1021/acs.langmuir.0c01154. 32659091

[ref28] Lu H.; Chen X.; Anthony J. E.; Johnson J. C.; Beard M. C. Sensitizing Singlet Fission with Perovskite Nanocrystals. J. Am. Chem. Soc. 2019, 141, 4919–4927. 10.1021/jacs.8b13562. 30821456

[ref29] Avakian P.; Merrifield R. E. Triplet Excitons in Anthracene Crystals-A Review. Mol. Cryst. 1968, 5, 37–77. 10.1080/15421406808082930.

[ref30] Tayebjee M. J. Y.; Clady R. G. C. R.; Schmidt T. W. The exciton dynamics in tetracene thin films. Phys. Chem. Chem. Phys. 2013, 15, 14797 10.1039/c3cp52609g. 23907164

[ref31] Stern H. L.; Cheminal A.; Yost S. R.; Broch K.; Bayliss S. L.; Chen K.; Tabachnyk M.; Thorley K.; Greenham N.; Hodgkiss J. M.; Anthony J.; Head-Gordon M.; Musser A. J.; Rao A.; Friend R. H. Vibronically coherent ultrafast triplet-pair formation and subsequent thermally activated dissociation control efficient endothermic singlet fission. Nat. Chem. 2017, 9, 1205–1212. 10.1038/nchem.2856. 29168494

[ref32] Wu T. C.; Thompson N. J.; Congreve D. N.; Hontz E.; Yost S. R.; Van Voorhis T.; Baldo M. A. Singlet fission efficiency in tetracene-based organic solar cells. Appl. Phys. Lett. 2014, 104, 193901 10.1063/1.4876600.

[ref33] Tkatchenko A.; Scheffler M. Accurate molecular van der Waals interactions from ground-state electron density and free-atom reference data. Phys. Rev. Lett. 2009, 102, 073005 10.1103/PhysRevLett.102.073005. 19257665

[ref34] Al-Saidi W. A.; Voora V. K.; Jordan K. D. An Assessment of the vdW-TS Method for Extended Systems. J. Chem. Theory Comput. 2012, 8, 1503–1513. 10.1021/ct200618b. 26596760

[ref35] Saidi W. A.; Choi J. J. Nature of the cubic to tetragonal phase transition in methylammonium lead iodide perovskite. J. Chem. Phys. 2016, 145, 144702 10.1063/1.4964094. 27782531

[ref36] Tomkiewicz Y.; Groff R. P.; Avakian P. Spectroscopic approach to energetics of exciton fission and fusion in tetracene crystals. J. Chem. Phys. 1971, 54, 4504–4507. 10.1063/1.1674702.

[ref37] Alvertis A. M.; Pandya R.; Muscarella L. A.; Sawhney N.; Nguyen M.; Ehrler B.; Rao A.; Friend R. H.; Chin A. W.; Monserrat B. Impact of exciton delocalization on exciton-vibration interactions in organic semiconductors. Phys. Rev. B 2020, 102, 081122(R) 10.1103/PhysRevB.102.081122.

[ref38] Haruyama J.; Sodeyama K.; Han L.; Tateyama Y. Termination dependence of tetragonal CH3NH3PbI 3 surfaces for perovskite solar cells. J. Phys. Chem. Lett. 2014, 5, 2903–2909. 10.1021/jz501510v. 26278097

[ref39] Al-Saidi W. A.; Rappe A. M. Density functional study of PbTiO3 nanocapacitors with Pt and Au electrodes. Phys. Rev. B 2010, 82, 155304 10.1103/PhysRevB.82.155304.

[ref40] Hlawacek G.; Teichert C. Nucleation and growth of thin films of rod-like conjugated molecules. J. Phys. Condens. Matter 2013, 25, 143202 10.1088/0953-8984/25/14/143202. 23478790

[ref41] Sun S.; Fang Y.; Kieslich G.; White T. J.; Cheetham A. K. Mechanical properties of organic-inorganic halide perovskites, CH_3_NH_3_PbX_3_ (X = I, Br and Cl), by nanoindentation. J. Mater. Chem. A 2015, 3, 18450–18455. 10.1039/C5TA03331D.

[ref42] Jhou Y. W.; Yang C. K.; Sie S. Y.; Chiu H. C.; Tsay J. S. Variations of the elastic modulus perpendicular to the surface of rubrene bilayer films. Phys. Chem. Chem. Phys. 2019, 21, 4939–4946. 10.1039/C8CP07062H. 30758010

[ref43] Wortman J. J.; Evans R. A. Young’s modulus, shear modulus and poisson’s ratio in Silicon and Germanium. J. Appl. Phys. 1965, 36, 153 10.1063/1.1713863.

[ref44] Chen Y.; Burgess T.; An X.; Mai Y. W.; Tan H. H.; Zou J.; Ringer S. P.; Jagadish C.; Liao X. Effect of a High Density of Stacking Faults on the Young’s Modulus of GaAs Nanowires. Nano Lett. 2016, 16, 1911–1916. 10.1021/acs.nanolett.5b05095. 26885570

[ref45] Abdi-Jalebi M.; Dar M. I.; Senanayak S. P.; Sadhanala A.; Andaji-Garmaroudi Z.; Pazos-Outón L. M.; Richter J. M.; Pearson A. J.; Sirringhaus H.; Grätzel M.; Friend R. H. Charge extraction via graded doping of hole transport layers gives highly luminescent and stable metal halide perovskite devices. Sci. Adv. 2019, 5, eaav2012 10.1126/sciadv.aav2012. 30793032 PMC6377269

[ref46] Bethe G. W.; Equation S.; Fujita T.; Noguchi Y. Revisiting the Charge-Transfer States at Pentacene / C 60 Interfaces with the. Materials 2020, 13, 2728 10.3390/ma13122728. PMC7345661 32560127

[ref47] Wieghold S.; Bieber A. S.; VanOrman Z. A.; Nienhaus L. Influence of Triplet Diffusion on Lead Halide Perovskite-Sensitized Solid-State Upconversion. J. Phy. Chem. Lett. 2019, 10, 3806–3811. 10.1021/acs.jpclett.9b01526. 31244265

[ref48] Saidi W. A.; Shadid W.; Castelli I. E. Machine-learning structural and electronic properties of metal halide perovskites using a hierarchical convolutional neural network. npj Comput. Mater. 2020, 6, 36 10.1038/s41524-020-0307-8.

