# Supplementary material for: Investigation of Singlet Fission–Halide Perovskite Interfaces
Source: Chem Mater. 2022 May 16;34(11):4865–75. doi: 10.1021/acs.chemmater.1c04310 (PMC9202303; doi:10.1021/acs.chemmater.1c04310)
Supplement: Supplementary file 1 — cm1c04310_si_001.pdf [file cm1c04310_si_001.pdf]

Supporting information: Investigation of singlet fission-halide perovskite interfaces

Alan R. Bowman,[1,2] Samuel D. Stranks,[1,3] and Bartomeu Monserrat*[1,4]

(*Email: bm418@cam.ac.uk)

[1)]*Cavendish Laboratory, Department of Physics, University of Cambridge, J.J. Thomson Avenue, Cambridge, CB3 0HE, U.K.*

[2)]*Current address: LNET-IGM-STI-EPFL, MED 1 2526, Station 9, CH-1015 Lausanne*

[3)]*Department of Chemical Engineering and Biotechnology, University of Cambridge, Philippa Fawcett Drive, Cambridge, CB3 0AS, U.K.*

[4)]*Department of Materials Science & Metallurgy, University of Cambridge, 27 Charles Babbage Road, Cambridge, CB3 0FS, U.K.*

(Dated: 6 May 2022)

Supporting Information A: List of experiments undertaken screening for triplet transfer

Fabrication can be split into two types: evaporation and solution processing. Tetracene and DPH were purchased from Sigma at the highest purity available (> 99.99 % and > 98 % respectively). All samples were fabricated on glass, which was cleaned in ultrasonic baths of acetone and then isopropanol prior to fabrication. All fabrication was carried out in nitrogen filled gloveboxes with oxygen levels of less than 10 ppm and water levels of less than 0.1 ppm. All samples were encapsulated immediately following fabrication, either by a two part or UV cured epoxy (Bluefixx). Halide perovskites marked 'in situ' were fabricated by the author, with other samples supplied by Rohit Prasanna and Matthew Klug[1,2] (see section A 2 for fabrication details). In all cases no triplet transfer was observed.

In general we found it was not straightforward to form a smooth interface between these materials, independent of the deposition method used. Instead, the two materials tended to separate, for example via the growth of pillars of the singlet fission material in evaporation (as in supporting information Figure S1b). While some solution processing methods allowed for smooth bilayer outer surfaces, it was still unclear whether a smooth interface between the two materials had been achieved.

A wide parameter space for singlet fission material deposition was explored (see following section for precise conditions) by using:

1. evaporation of the organic on the halide perovskite, with evaporation rates from 0.5 Ås^{-1} to 17 Ås^{-1} and deposition thicknesses from 7.5 nm to 150 nm

2. spin coating the organic on the halide perovskite, with singlet fission materials dissolved in chlorobenzene (which halide perovskites are not soluble in), spinning speeds from 2000 to 4000 rotations per minute and sample annealing times from 0 to 30 minutes (prior to encapsulation)

3. drop casting the organic on the halide perovskite, with different solution concentrations deposited (though this resulted in extremely thick organic films in all cases, so was not significantly investigated)

4. spin-coating the halide perovskite on singlet fission materials (noting this resulted in much of the singlet fission material being removed from the substrate)

5. spin-coating the singlet fission material and halide perovskite at once to attempt to form heterojunctions.

Full details of deposition conditions are now briefly listed.

1. Evaporation

All evaporations were carried out at a pressure of $\sim 1 \times 10^{-5}$ mbar or lower. Tetracene was evaporated at a temperature of 120°C, DPH at110°C, CBP at 190°C and C_{60} at 400°C (all Sigma). Note for DPH evaporations thicknesses and evaporation rates are nominal as tetracene tooling factors were used. DPH is a heavier molecule so these should be regarded as upper bounds.

1. 30 nm of tetracene was evaporated at a rate of 0.5 $\text{Å}s^{-1}$ on $FAPb_{0.5}Sn_{0.5}I_3$ samples, with a small proportion of the lead and tin replaced with x=0 %, 2 %, 5 % and 10 % Ca, Mg, Sr, Zn, Co and Ni (where x is defined as in Bowman and co-workers[3]). Halide perovskite samples were $\sim$ 200 nm thick.

2. 30 nm of tetracene was evaporated at a rate of 0.5 $\text{Å}s^{-1}$ on x=0 %, 2 % and 10 % Mg, Sr and Co samples. Halide perovskite samples were $\sim$ 200 nm thick.

3. 60 nm of tetracene was evaporated at a rate of 1 $\text{Å}s^{-1}$ on x=0 %, 2 % and 10 % Co and Zn samples. Halide perovskite samples were $\sim$ 200 nm thick.

4. 60 nm of tetracene was evaporated at a rate of 0.7 $\text{Å}s^{-1}$ on $MA_yFA_{1-y}Sn_{0.75}Pb_{0.25}I_3$ samples, with y=0, 0.2, 0.4, 0.6, 0.8 and 1. Halide perovskite samples were a range of thicknesses around 300 nm.

5. 150 nm of DPH was evaporated at a rate of 0.5 $\text{Å}s^{-1}$ on x=0 %, 2 %, 5 % and 10 % Zn samples. Halide perovskite samples were $\sim$ 200 nm thick.

6. 20/150/150 nm of DPH was evaporated at a rate of 0.5/0.8/7.0 $\text{Å}s^{-1}$ on $\sim$ 200 nm thick $Cs_zFA_{1-z}Sn_{0.5}Pb_{0.5}I_3$ with z=0, 0.05, 0.1, 0.15 and 0.2, for x=0 % and 5 % Zn.

7. 150 nm of DPH was evaporated at a rate of 6.5/0.5/2.5/17 $\text{Å}s^{-1}$ on $\sim$ 100 nm thick $Cs_zFA_{1-z}Sn_{0.75}Pb_{0.25}I_3$, z= 0, 0.05, 0.1, 0.15, 0.2, $MA_yFA_{1-y}Sn_{0.75}Pb_{0.25}I_3$, y=0.4, 0.8, 1.0 and in situ fabricated $FA_{0.75}Cs_{0.25}Sn_{0.75}Pb_{0.25}I_3$.

8. 5.2 nm of C_{60} was evaporated on $MA_yFA_{1-y}Sn_{0.75}Pb_{0.25}I_3$ with y=0, 0.8 and 1 at a rate of 0.4 Ås^{-1} (as C_{60} is known to separate triplets from singlet fission materials). On these samples, and equivalent samples samples without C_{60} deposited, 5/50/500/500 nm of DPH/DPH/tetracene/DPH was evaporated at a rate of 15 Ås^{-1} (noting that higher evaporation rates was observed to reduce the formation of pillars). Halide perovskites were ∼ 100 nm thick.

9. x=0 % and x=5 % Zn samples of ∼ 200 nm and ∼ 50-100 nm thicknesses had either DPH, or DPH followed by CBP evaporated on them. For the former, 100 nm of DPH was evaporated at 1 Ås^{-1}, while for the latter 7.5 nm DPH was evaporated at 1.2 Ås^{-1} followed by 100 nm CBP at 1 Ås^{-1}. This was with the suggestion of transferring singlet excitons from a high-bandgap absorber (CBP) to a thin layer of singlet fission material (DPH).

10. 7.5 nm of DPH was evaporated at a rate of 5 Ås^{-1} on ∼ 500 nm and ∼ 100 nm thickness $FASn_{0.75}Pb_{0.25}I_3$ and $MASn_{0.75}Pb_{0.25}I_3$ samples. On half of these samples (four of each were made) 200 nm of CBP was evaporated at a rate of 1.2 Ås^{-1}, again with the suggestion of transferring singlet excitons from a high-bandgap absorber (CBP) to a thin layer of singlet fission material (DPH).

2. Solution processing

All solution processing was carried out in nitrogen filled gloveboxes with < 10ppm O_2 and < 1ppm H_2O.

All 'in situ' fabricated low-bandgap $FA_{0.75}Cs_{0.25}Sn_{0.75}Pb_{0.25}I_3$ halide perovskites samples were fabricated from stock solutions of PbI_2 (0.28 M), SnI_2 (0.83 M), SnF_2 (0.16 M), FAI (0.83 M) and CsI (0.28 M) in a 65:35 solution of DMF:DMSO (all Sigma). In all cases films were spin coated: 30 μL of the solution was deposited on a substrate which was spun at 4000 rotations per minute for 30 s, with a gas quench applied from 15 s. Samples were annealed at 100°C for 15 minutes. Solution processed experiments undertaken are as follows:

1. A stock solution for in situ fabrication was diluted to 10 % of normal concentration and low-bandgap halide perovskites were deposited (achieving very thin films). 0.03/0.07/0.18 M DPH in chlorobenzene was prepared at 25/50/80°C (corresponding to the satulation limit at each temperature) and statically deposited on the halide perovskite. Four of each sample

were made, half of which were spun at 1000 rotations per minute for 20 s (i.e. spin-coating and drop casting was carried out). One of each sample type was annealed at 100°C for 15 minutes while the others were left to air dry.

2. Bulk heterojunctions were spin-coated in situ. A $FA_{0.75}Cs_{0.25}Sn_{0.75}Pb_{0.25}I_3$ precursor solution was split into four. The first was used as a control solution, the second had 0.04 M DPH dissolved into it (DPH's solubility limit in these solutions at room temperature), the third was diluted by 50 % and then had 0.04 M DPH dissolved into it and the last was heated to 70°C and had 0.13 M DPH dissolved into it.

Supporting Information B: Other experimental methods

1. Atomic force microscopy

Atomic force microscopy (AFM) was carried out using an Asylum Research MFP-3D atomic force microscope in non-contact AC mode. 0th order flattening and 1st order plane fits were applied to all data. All measurements and data processing were carried out on Asylum Research AFM Software version 15.

2. Photoluminescence

In photoluminescence measurements samples were excited by a continuous wave temperature controlled Thorlabs 405 nm laser. The emission was recorded using an Andor IDus DU420A silicon detector for lead-only samples, otherwise an Andor IDus DU490A InGaAs detector.

3. Time resolved photoluminescence

Time-resolved PL spectra were recorded using time correlated single photon counting. Specifically, a Fianium WhiteLase High Power Supercontinuum laser (LW-SC-IR-8) produced pulses which were passed through bandpass filters with 10 nm bandwidth, centered at either 450 nm or 650 nm, as discussed in the main text. In all cases a 2 MHz repetition rate was used. The beam was focused on to the sample. Emitted light was passed through a relevant longpass or bandpass filter to focus on light at relevant wavelengths (as discussed in main text). This light was focused on an

MPD PDM single-photon avalanche photodiode, giving the time resolved photoluminescence. In all cases the recorded signal was less than 1 % of the number of incident photons.

Supporting Information C: Computational details

We carried out geometry optimisations with the density functional theory (DFT) code CASTEP[4] with on-the-fly generated ultra-soft pseudopotentials. Spin-orbit coupling was not included in our geometry optimisations as we found it had a small effect relative to the additional computational effort: the lattice parameter of cubic $CsPbI_3$ only changed from 6.155Å to 6.185Å with the inclusion of spin-orbit coupling. We used a cutoff energy of 400 eV in all geometry optimisations. Van der-Waals semi-empirical corrections were required for all calculations to correctly reproduce tetracene's geometry and electronic structure. For primitive tetracene and halide perovskite unit cells we used a Monkhorst-Pack $\vec{k}$-point grid of $5 \times 5 \times 5$, while for larger cells (e.g. two repeating tetracene and three repeating halide perovskite units in the non-vacuum direction) we used a commensurate reduced number of $\vec{k}$-points in periodic directions (with a minimum of 2 $\vec{k}$-points being used in these directions), while in the non-periodic direction only the Γ point was sampled. In all supercells with a vacuum, we converged the vacuum size to be large enough to not affect results. This corresponded to a vacuum at least 0.8 times the size of the unit cell for $CsPbI_3$ and at least the same length as the unit cell size for tetracene. The same cutoff energies and $\vec{k}$-point grids were used for density of states (DOS) and projected-DOS (PDOS) calculations. For density of states calculations including spin-orbit coupling we used a cutoff energy of 500eV, a Monkhorst-Pack $\vec{k}$-point grid of $5 \times 5 \times 5$ for SCF calculations, a Monkhorst-Pack $\vec{k}$-point grid of $6 \times 6 \times 6$ for spectral calculations and CASTEP's norm-conserving pseudopotentials. For calculations of a tetracene molecule on a halide perovskite surface we fixed the lattice parameters, while for thin-film interfaces lattice parameters were allowed to vary freely.

We used the DFT code QUANTUM ESPRESSO and post-DFT code YAMBO to calculate electronic and excitonic states[5–7]. In QUANTUM ESPRESSO we used $\vec{k}$-point grids of $6 \times 6 \times 6$ (so the Γ and R points were both directly sampled), with a cutoff energy of 680 eV (50 Ry). Norm-conserving Vanderbilt pseudopotentials, taken from the Schlipf-Gygi norm-conserving pseudopotential library, were employed in these computations as they are optimised for subsequent YAMBO calculations[8–10]. In all our YAMBO calculations, parameters were converged to give results to an accuracy of at least 0.05 eV. To aid with YAMBO calculations at full interfaces, we sometimes ig-

nored the non-local commutator (noted in text), which was found to affect calculations on tetracene and halide perovskite only minimally (changing energies by <0.05 eV), and for toy models we reduced the maximum size of reciprocal lattice vectors (corresponding to the cut-off energy used in DFT calculations) with respect to that in QUANTUM ESPRESSO calculations (which affected the accuracy of calculations to < 0.01 eV).

All our visualisations were carried out with a combination of C2X and VESTA[11,12].

Supporting Information D: Bulk $CsPbI_3$

Iodine and lead are the main contributors to the valence and conduction bands in experimental halide perovskites at room temperature[16]. However, in the fully relaxed cubic $CsPbI_3$, we found the nature of the valence and conduction bands is inverted at the DFT-level when including spin-orbit coupling (when using PBE with TS correction). We found increasing the halide perovskite's lattice parameter by 2 % or more allows for correct band ordering at the DFT-level (with iodine/lead being the main contributor to the valence/conduction band), as plotted in Figure S2 a and b.

We carried out a one shot G_0W_0 correction to the cubic halide perovskite's band structure. While G_0W_0 corrections do not give halide perovskites' bandgaps with full accuracy, calculations beyond G_0W_0 are too computationally intensive to carry out when modelling interfaces[17,18]. In order to have correct band ordering for calculations, we carried out G_0W_0 calculations on structures with the lattice parameter 2 % to 5 % larger than the relaxed structure. The DFT- and G_0W_0-level bandgaps for these cells (with spin-orbit coupling) are shown in Figure S2c. From these results we extrapolated the approximate bulk bandgap of relaxed $CsPbI_3$ as 0.98 eV. This is close to the DFT-level bandgap without spin-orbit coupling of 1.09 eV, suggesting the halide perovskite's electronic structure without spin-orbit coupling approximately models results from post-DFT methods with spin-orbit coupling included. We use this to approximately model electronic states at interfaces in subsequent sections (and calculate approximate errors associated with this).

Supporting Information E: G_0W_0 for different tetracene/halide perovskite electronic states and scissor corrections

The effect of G_0W_0 corrections of different isolated tetracene and halide perovskite models, and small interfaces, are presented in Table IV. All models presented are in approximate agreement, with the tetracene valence band being increased in energy by $\sim$ 1eV more than the halide perovskite valence band following a G_0W_0 correction, and the tetracene conduction band being increased by at least 1.5eV more than the halide perovskite conduction band.

For the CsI terminated toy model, the offset between the halide perovskite and tetracene's valence band is 0.97eV without spin-orbit coupling and 0.78eV with spin-orbit coupling. This suggests (alongside the results in Table IV) that the qualitative results drawn from PDOS calculations in sections VI and VII (without spin-orbit coupling) would still be valid with the inclusion of spin-orbit coupling and subsequent G_0W_0 corrections, with tetracene valence band state being further increased in energy with respect to the halide perovskite valence band state.

We present tetracene's G_0W_0 bandgap, singlet and triplet energies for the toy interfaces, bulk tetracene and tetracene in a vacuum in Table V. There are three models for tetracene in a vacuum – geometry relaxed, and the same geometries as used at both PbI_2 and CsI terminated toy models. Finally, we present scissor shifts applied in the main text in Table VI.

Supporting Information F: G_0W_0 calculations for PbI_2 terminated surfaces

In Figure S6a and b we plot average exciton electron and hole position for PbI_2 termination (for lowest energy triplet and singlet states). We note this is not the electron wavefunction with the hole fixed in one place, as was presented in Figure 2. Here the average electron wavefunctions are found to have a small contribution within the halide perovskite (noting isosurfaces are only on tetracene up to the 88 % probability surface). However, within our simulations tetracene makes up only a small region of the simulation cell and therefore there are contributions to the average electron position both from the hole being in vacuum and on the halide perovskite. To this end, we plot the electron charge density in Figure S6c and d for the hole (grey sphere) fixed on a tetracene molecule. These plots reveal that when the hole is fixed on a tetracene molecule, the electron also is. We observed this for the hole being fixed at many different locations within tetracene. These results again demonstrate that tetracene's excitonic states are strongly localised, even at an

interface. This is also suggestive that electrons in the halide perovskite may transfer into tetracene and then become localised within the organic.

Functional	Lattice parameter
Experimental[13]	$\vec{a} = (7.9, 0.0, 0.0)$
(no temperature stated,	$\vec{b} = (0.39, 6.02, 0.0)$
assumed room temperature)	$\vec{c} = (-5.33, -2.08, 12.26)$
LDA	$\vec{a} = (7.33, 0.0, 0.0)$
	$\vec{b} = (0.48, 6.01, 0.0)$
	$\vec{c} = (-5.46, -2.19, 11.93)$
Root mean square difference with experiment	0.23
PBE + TS	$\vec{a} = (7.68, 0.0, 0.0)$
	$\vec{b} = (0.45, 6.02, 0.0)$
	$\vec{c} = (-5.36, -2.19, 12.09)$
Root mean square difference with experiment	0.10
PBE + G06[14]	$\vec{a} = (7.35, 0.0, 0.0)$
	$\vec{b} = (0.55, 6.14, 0.0)$
	$\vec{c} = (-5.88, -2.40, 11.91)$
Root mean square difference with experiment	0.31
PBE + JCHS[15]	$\vec{a} = (6.79, 0.0, 0.0)$
	$\vec{b} = (0.65, 5.90, 0.0)$
	$\vec{c} = (-5.76, -2.68, 11.36)$
Root mean square difference with experiment	0.54
PBESOL + TS	$\vec{a} = (7.53, 0.0, 0.0)$
	$\vec{b} = (0.47, 6.08, 0.0)$
	$\vec{c} = (-5.57, -2.23, 12.04)$
Root mean square difference with experiment	0.17

TABLE I. Lattice parameters obtained from geometry optimisations of bulk tetracene with different exchange correlation functionals, alongside experimental result. $\vec{a}$, $\vec{b}$ and $\vec{c}$ are the lattice vectors in Cartesian coordinates, and all lengths are in Å. PBE and PBESOL functionals did not lead to a relaxed geometries without Van der Waals corrections.

Number of repeating units	Pb-I	Cs-Cs	Perpendicular lattice parameter
CsI termination			
1	3.19	4.89	6.11
2	3.24	5.19	6.13
3	3.06	5.59	6.13
4	3.07	5.79	6.13
5	3.04	5.99	6.14
7	3.07	6.13	6.14
8	3.07	6.15	6.14
PbI_2 termination			
1	3.06	N/A	6.22
2	3.08	5.79	6.23
3	3.08	5.93	6.20
4	3.07	6.10	6.20
5	3.08	6.10	6.18
6	3.08	6.17	6.20
7	3.09	6.18	6.18
8	3.08	6.14	6.17

TABLE II. Inter-atomic distances in the centre of a halide perovskite slab, in the vacuum direction, for both Cs-Cs and Pb-I distances, and lattice parameters perpendicular to the vacuum, for both CsI and PbI_2 terminations. All values presented are in Å. We note that the relaxed bulk cubic lattice parameter is 6.16 Å.

	CsI termination	PbI_2 termination
Cut 1 no rotation		
Strain in x direction (%)	-0.3	0.9
Strain in y direction (%)	1.4	2.6
Shear strain (%)	7.5	7.5
Cut 1 with rotation		
Strain in x direction (%)	1.7	2.8
Strain in y direction (%)	1.4	2.6
Shear strain (%)	12.3	12.3
Cut 2		
Strain in x direction (%)	1.4	2.6
Strain in y direction (%)	-6.1	-4.87
Shear strain (%)	5.1	5.1

TABLE III. The normal and shear strain for each geometry presented in Figure S3, relative to the relaxed halide perovskite slab in a vacuum.

Model	$E_{Tc,VB,shift} - E_{P,VB,shift}$ (eV)	$E_{Tc,CB,shift} - E_{P,CB,shift}$ (eV)
Bulk systems (no SO)	1.53	1.76
Bulk systems (with SO)	1.38	1.88
Bulk P and Tc in vacuum (no SO)	0.83	1.81
Bulk P and Tc in vacuum (with SO)	0.69	1.92
CsI terminated toy model (no SO)	1.37	1.20
CsI terminated toy model (with SO)	1.30	1.40
PbI_2 terminated toy model (no SO)	1.37	1.08

TABLE IV. The energy difference in G_0W_0 corrections between tetracene and halide perovskite states, for different bulk and interface models. Here $E_{Tc,VB,shift} = E_{Tc,VB,G0W0} - E_{Tc,VB,DFT}$, the difference in energy between tetracene's (Tc) valence band energy at G_0W_0 and DFT levels. Other subscripts carry similar meanings, with P corresponding to halide perovskite, CB to conduction band and SO to spin-orbit coupling.

Model	$E_{g,Tc,G0W0}$ (eV)	$E_{S,Tc}$ (eV)	$E_{T,Tc}$ (eV)
Bulk tetracene	2.62	2.08	1.20
Tetracene in vacuum (relaxed)	3.40	2.04	1.14
Tetracene in vacuum (CsI toy geometry)	3.28	1.92	1.11
Tetracene in vacuum (PbI_2 toy geometry)	3.30	1.94	1.12
CsI terminated toy model (no SO)	2.13	1.91	1.12
CsI terminated toy model (with SO)	2.64	1.94	1.17
PbI_2 terminated toy model (no SO)	2.24	1.87	1.08

TABLE V. Tetracene's G_0W_0 level bandgap ($E_{g,Tc,G0W0}$) and lowest singlet ($E_{S,Tc}$) and triplet ($E_{T,Tc}$) energies are presented for bulk tetracene, a layer of (cut 1) tetracene in a vacuum (both relaxed and for the geometry used in toy models) and for toy models with scissor corrections applied to correctly reproduce tetracene's electronic states. Here SO means spin-orbit coupling.

Model	ΔE_g (eV)	M_c	M_v
CsI termination			
Physically correct scissor correction	1.88	1.20	1.23
Alternative scissor correction	1.30	2.05	1.23
PbI_2 termination			
Physically correct scissor correction	1.76	1.11	1.22
Alternative scissor correction	1.00	2.75	1.22

TABLE VI. Scissor corrections applied to the toy interface models are presented. Here ΔE_g is the value added to the DFT-level bandgap and M_c and M_v are the gradient corrections applied to the DFT-level conduction and valence bands (for more details see YAMBO WIKI[19]). Physically correct scissor corrections are those found from fitting G_0W_0 calculations (c.f Figure 6) and alternative corrections are those applied to produce charge transfer states lower in energy than tetracene's lowest energy triplet.

Supporting Information G: Additional supporting information figures

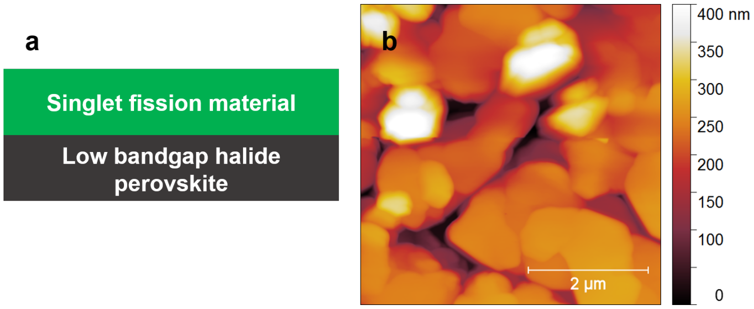

FIG. S1. a) A schematic of a singlet fission/halide perovskite bilayer. b) an atomic force microscopy image of the surface of evaporated 1,6-Diphenyl-1,3,5-hexatriene (DPH).

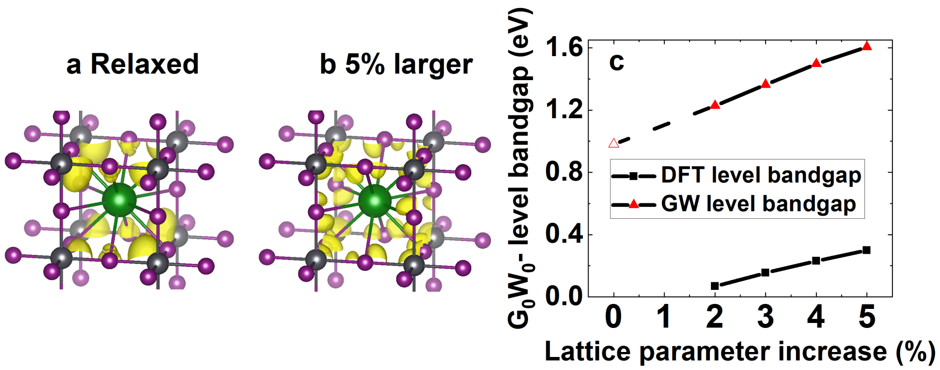

FIG. S2. a) and b) plot the valence band charge density (at the band edge) of cubic $CsPbI_3$ when fully relaxed, and with the lattice parameter increased by 5 % (as marked on figure). The latter gives the experimental halide perovskite electronic structure with four regions of high charge density along a lattice parameter. As discussed in Supporting Information D, it was not possible to carry out G_0W_0 corrections on the relaxed halide perovskite. Instead, in c) G_0W_0 calculations for the lattice parameter increased between 2 % and 5 % are presented, which allows for an estimation of the relaxed G_0W_0 bandgap, as marked by the dashed line. All calculations include spin-orbit coupling.

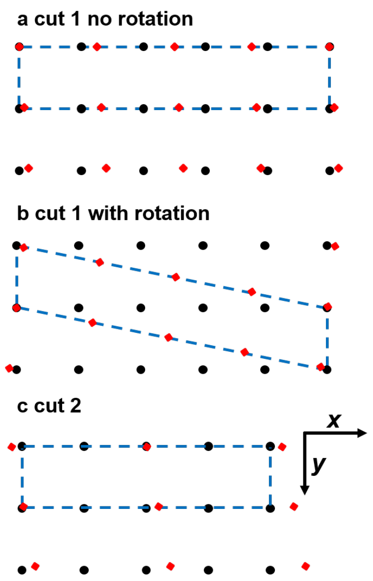

FIG. S3. Commensurate tetracene/$CsPbI_3$ unit cells. Black circles and red diamonds are halide perovskite and tetracene lattice points respectively. The three commensurate cells, ‘cut 1 no rotation’, ‘cut 1 with rotation’ and ‘cut 2’ are presented in a), b) and c) respectively, with the dashed blue line representing one repeating unit.

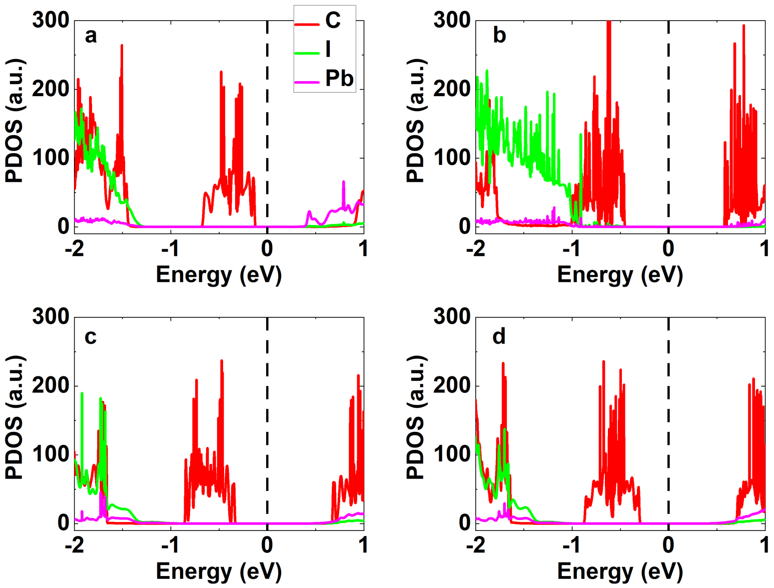

FIG. S4. PDOS, without spin-orbit coupling, are presented for relaxed interfaces of: cut 1 with rotation, PbI_2 termination; cut 2 PbI_2 termination; cut 1 no rotation, CsI termination; and cut 1 with rotation, CsI termination, in a), b), c) and d) respectively. Dashed vertical lines mark the Fermi level.

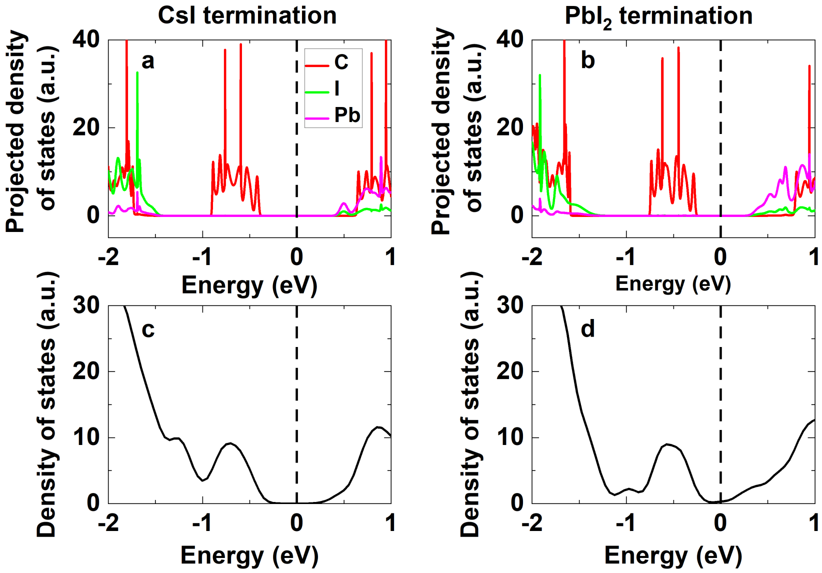

FIG. S5. a) and b) present the PDOS (without spin-orbit coupling) for CsI and PbI_2 terminated toy models. The density of states with spin-orbit coupling for the same models are shown in c) and d). In all plots the dashed line corresponds to the highest occupied level. Different smearing parameters have been used in plots with and without spin-orbit coupling.

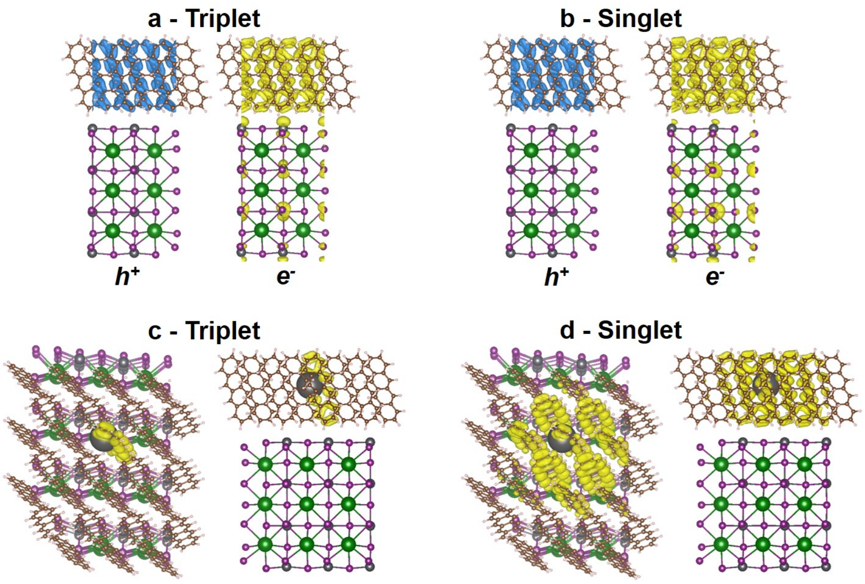

FIG. S6. Average hole (h^+) and electron (e^-) charge densities for the lowest energy triplet and singlet excitons for the PbI_2 terminated toy model are plotted in a) and b), for the G_0W_0 correction which correctly reproduces tetracene's electronic states. The electron density of the same states with the hole (grey sphere) fixed on a tetracene molecule are plotted in c) and d), for two different unit cell orientations in each case. Isosurface marks the 95 % probability boundary in all plots.

REFERENCES

[1]R. Prasanna, A. Gold-Parker, T. Leijtens, B. Conings, A. Babayigit, H. G. Boyen, M. F. Toney, and M. D. McGehee, "Band Gap Tuning via Lattice Contraction and Octahedral Tilting in Perovskite Materials for Photovoltaics," J. Am. Chem. Soc. **139**, 11117–11124 (2017).

[2]M. T. Klug, A. Osherov, A. A. Haghighirad, S. D. Stranks, P. R. Brown, S. Bai, J. T.-W. Wang, X. Dang, V. Bulović, H. J. Snaith, and A. M. Belcher, "Tailoring metal halide perovskites through metal substitution: influence on photovoltaic and material properties," Energy Environ. Sci. **10**, 236–246 (2017).

[3]A. R. Bowman, M. T. Klug, T. A. S. Doherty, M. D. Farrar, S. P. Senanayak, B. Wenger, G. Divitini, E. P. Booker, Z. Andaji-Garmaroudi, S. Macpherson, E. Ruggeri, H. Sirringhaus, H. J. Snaith, and S. D. Stranks, "Microsecond Carrier Lifetimes, Controlled p-Doping, and Enhanced Air Stability in Low-Bandgap Metal Halide Perovskites," ACS Energy Lett. **4**, 2301–2307 (2019).

[4]S. J. Clark, M. D. Segall, C. J. Pickard, P. J. Hasnip, M. I. J. Probert, K. Refson, and M. C. Payne, "First principles methods using CASTEP," Zeitschrift für Krist. **220**, 567–570 (2005).

[5]P. Giannozzi, S. Baroni, N. Bonini, M. Calandra, R. Car, C. Cavazzoni, D. Ceresoli, G. L. Chiarotti, M. Cococcioni, I. Dabo, A. Dal Corso, S. De Gironcoli, S. Fabris, G. Fratesi, R. Gebauer, U. Gerstmann, C. Gougoussis, A. Kokalj, M. Lazzeri, L. Martin-Samos, N. Marzari, F. Mauri, R. Mazzarello, S. Paolini, A. Pasquarello, L. Paulatto, C. Sbraccia, S. Scandolo, G. Sclauzero, A. P. Seitsonen, A. Smogunov, P. Umari, and R. M. Wentzcovitch, "QUANTUM ESPRESSO: A modular and open-source software project for quantum simulations of materials," J. Phys. Condens. Matter **21**, 395502 (2009).

[6]P. Giannozzi, O. Andreussi, T. Brumme, O. Bunau, M. Buongiorno Nardelli, M. Calandra, R. Car, C. Cavazzoni, D. Ceresoli, M. Cococcioni, N. Colonna, I. Carnimeo, A. Dal Corso, S. De Gironcoli, P. Delugas, R. A. DiStasio Jr, A. Ferretti, A. Floris, G. Fratesi, G. Fugallo, R. Gebauer, U. Gerstmann, F. Giustino, T. Gorni, J. Jia, M. Kawamura, H.-Y. Ko, A. Kokalj, E. Kucukbenli, M. Lazzeri, M. Marsili, N. Marzari, F. Mauri, N. L. Nguyen, N. L. Nguyen, H.-V. Nguyen, A. Otero-de-la Roza, L. Paulatto, S. Ponce, D. Rocca, R. Sabatini, B. Santra, M. Schlipf, A. P. Seitsonen, A. Smogunov, I. Timrov, T. Thonhauser, P. Umari, N. Vast, X. Wu, and S. Baroni, "Advanced capabilities for materials modelling with Quantum ESPRESSO," J. Phys. Condens. Matter **29**, 465901 (2017).

[7]A. Marini, C. Hogan, M. Grüning, and D. Varsano, "Yambo: An ab initio tool for excited state calculations," Comput. Phys. Commun. **180**, 1392–1403 (2009).

[8]D. R. Hamann, "Optimized norm-conserving Vanderbilt pseudopotentials," Phys. Rev. B - Condens. Matter Mater. Phys. **88**, 1–10 (2013).

[9]M. Schlipf and F. Gygi, "Optimization algorithm for the generation of ONCV pseudopotentials," Comput. Phys. Commun. **196**, 36–44 (2015).

[10]P. Scherpelz, M. Govoni, I. Hamada, and G. Galli, "Implementation and Validation of Fully Relativistic GW Calculations: Spin-Orbit Coupling in Molecules, Nanocrystals, and Solids," J. Chem. Theory Comput. **12**, 3523–3544 (2016).

[11]M. J. Rutter, "C2x: A tool for visualisation and input preparation for CASTEP and other electronic structure codes," Comput. Phys. Commun. **225**, 174–179 (2018), arXiv:1712.06640.

[12]K. Momma and F. Izumi, "VESTA 3 for three-dimensional visualization of crystal, volumetric and morphology data," J. Appl. Crystallogr. **44**, 1272–1276 (2011).

[13]R. B. Campbell, J. M. Robertson, and J. Trotter, "The crystal structure of hexacene, and a revision of the crystallographic data for tetracene," Acta Crystallogr. **15**, 289–290 (1962).

[14]S. Grimme, "Semiempirical GGA-Type Density Functional Constructed with a Long-Range Dispersion Correction," J. Comput. Chem. **27**, 1787–1799 (2006).

[15]P. Jurecka, J. Cerny, P. Hobza, and D. R. Salahub, "Density Functional Theory Augmented with an Empirical Dispersion Term. Interaction Energies and Geometries of 80 Noncovalent Complexes Compared with Ab Initio Quantum Mechanics Calculations," J. Comput. Chem. **28**, 555–569 (2007).

[16]J. Haruyama, K. Sodeyama, L. Han, and Y. Tateyama, "Termination dependence of tetragonal $CH_3NH_3PbI_3$ surfaces for perovskite solar cells," J. Phys. Chem. Lett. **5**, 2903–2909 (2014).

[17]M. R. Filip and F. Giustino, "GW quasiparticle band gap of the hybrid organic-inorganic perovskite $CH_3NH_3PbI_3$: Effect of spin-orbit interaction, semicore electrons, and self-consistency," Phys. Rev. B - Condens. Matter Mater. Phys. **90**, 1–10 (2014).

[18]L. Leppert, T. Rangel, and J. B. Neaton, "Towards predictive band gaps for halide perovskites: Lessons from one-shot and eigenvalue self-consistent GW," Phys. Rev. Mater. **3**, 103803 (2019).

[19]Yambo, "http://www.yambo-code.org/wiki," (2021).
